# The intentions of information sources can affect what information people think qualifies as true

**DOI:** 10.1038/s41598-023-34806-4

**Published:** 2023-05-12

**Authors:** Isaac J. Handley-Miner, Michael Pope, Richard Kenneth Atkins, S. Mo Jones-Jang, Daniel J. McKaughan, Jonathan Phillips, Liane Young

**Affiliations:** 1 Department of Psychology and Neuroscience, Boston College, Chestnut Hill, MA 02467 USA; 2 Department of Philosophy, Boston College, Chestnut Hill, MA 02467 USA; 3 Department of Communication, Boston College, Chestnut Hill, MA 02467 USA; 4 Program in Cognitive Science, Dartmouth College, Hanover, NH 03755 USA

**Keywords:** Psychology, Human behaviour

## Abstract

The concept of truth is at the core of science, journalism, law, and many other pillars of modern society. Yet, given the imprecision of natural language, deciding what information should count as true is no easy task, even with access to the ground truth. How do people decide whether a given claim of fact qualifies as true or false? Across two studies (N = 1181; 16,248 observations), participants saw claims of fact alongside the ground truth about those claims. Participants classified each claim as true or false. Although participants knew precisely how accurate the claims were, participants classified claims as false more often when they judged the information source to be intending to deceive (versus inform) their audience, and classified claims as true more often when they judged the information source to be intending to provide an approximate (versus precise) account. These results suggest that, even if people have access to the same set of facts, they might disagree about the truth of claims if they attribute discrepant intentions to information sources. Such findings may shed light on the robust and persistent disagreements over claims of fact that have arisen in the “post-truth era”.

## Introduction

Although people employ the concept of truth daily when sifting through the deluge of information encountered in modern society, classifying something as true or false is no easy task. Natural language is vague and rife with approximations. People regularly contend with classifying claims as true or false in the context of numerical estimates (e.g., rounded numbers), indefinite adjectives (e.g., “few,” “many”), generalizations (e.g., references to entire groups), and omissions (e.g., claims lacking important context). Given these challenges, how do people decide whether to classify claims of fact as true or as false, even if they know exactly how accurate the claims are? Imagine, for example, a scientific paper is published that predicts a novel virus will infect 1.21% of the country’s population in the next month. A news headline reports, “Scientific paper predicts new virus will infect 1% of the nation’s population by next month.” Even if people knew the results of the original scientific paper, they might disagree about the veracity of this headline. Given the imprecision of natural language, what factors influence people’s decisions about whether a piece of information qualifies as true or false?

One possibility is that people decide whether to classify claims as true by considering only the objective accuracy of the claims. Arguably, this is how matters of truth and falsity are often discussed in popular discourse—as if the labels “true” and “false” should correspond to the objective discrepancy between an alleged state of the world and the actual state of the world. If this were the case, people evaluating the example above should appeal only to the discrepancy of 0.21% between the report and the original scientific finding. We propose an alternative possibility, however: people might additionally consider features of the source of the claim—specifically, the source’s intentions—when deciding whether to classify a claim as true. If this were the case, people evaluating the example above might be swayed by whether they thought the news source intended to downplay the severity of the forthcoming contagion.

Decades of work on human social cognition has shown that people consider others’ intentions when evaluating their actions, including their speech acts^1–4^. A seminal paper by Grice^5^ proposed that communicators generally adhere to a Cooperative Principle, consisting of a constellation of norms (dubbed Gricean Maxims) that allow listeners to successfully infer the intended meaning of utterances. Interpreting others’ speech acts in light of their intentions allows forms of speech like sarcasm to be interpreted correctly. For example, if someone makes a crude remark, and an observer comments, “wow, he’s a real gentleman,” listeners do not interpret that statement literally because they infer the speaker’s intentions. Additionally, people form attitudes about everything from consumer goods to political views depending on the credibility people ascribe to relevant information sources^6,7^. One key way people assess credibility, and make other similar epistemic and moral judgments, is by inferring the intentions of others^8^. For example, even children 5–6 years of age appear to believe the testimony of an information source less if the source tried to be deceptive rather than informative in a previous task^9^. Moreover, people’s false statements have been shown to be considered more morally bad and more of a lie when the statements were intended to harm, rather than help, the target of the statement^10,11^. In short, people regularly infer and account for intent in order to interpret the meaning of others’ speech and evaluate the reliability of information sources.

Building on these findings, this paper tests whether attributions of intent affect what information people think qualifies as true. Specifically, do the intentions people attribute to information sources affect whether people classify claims as true or false—even when people know precisely how accurate or inaccurate those claims are?

What information people believe to be true has received increased scrutiny and, arguably, taken on even greater importance in recent years during what some have dubbed the “post-truth era.” Building on decades of psychology research on belief formation, a burgeoning literature on misinformation has sought to identify the circumstances in which people buy into and share inaccurate information. Recent reviews cover this work in depth^12–15^, but, in brief, evidence suggests that people can believe false information for a whole host of reasons, including relying on familiarity and processing fluency as cues of truth^16^, seeking congruence with pre-existing worldviews^14,17,18^, relying on intuitive and non-deliberative thinking^19–21^, and using flawed cues of source credibility such as one’s perceived similarity with a source^22–25^. One high-level theoretical account argues that motivated reasoning is largely to blame for endorsement of misinformation and partisan disagreement over the veracity of claims. This view suggests that people often form beliefs in service of non-epistemic goals, such as aligning attitudes with political ingroup members^15,26–29^. Meanwhile, another dominant account argues that lack of critical reasoning is the primary culprit for the spread and influence of misinformation. This view contends that many people do not deliberate sufficiently on information they encounter before endorsing it^14,30^. Although these two high-level accounts continue to be debated, the misinformation literature has produced mountains of research describing when and why people are likely to believe a given piece of information is true.

However, unlike most existing work on misinformation and belief formation, this paper does not assess how people *discern* true versus false information. Rather, this paper seeks to understand what people think even *qualifies* as true versus false information. In other words, even when the truth is known, how do people decide whether to consider a claim to be true or false? Understanding what people think qualifies as true and false is neglected in the misinformation literature and broader psychology literature, but may be critical to making sense of and intervening in the post-truth era. For example, if situational factors such as perceived intentions of an information source affect what people consider to be true, it could be that some contemporary disagreements over claims of truth are not actually about the objective accuracy of a news report, political claim, or scientific finding, but about the intentions of the journalist, politician, or scientist. Additionally, it could suggest that getting people to “agree on the facts” may be insufficient for resolving disagreements over the truth, and that interventions should also focus on boosting trust in the intentions of society’s most important information sources. Moreover, it can shed light on how people react to intentionally misleading information, a particularly important topic given that information that is not completely false can still be misleading, and possibly even more convincing than complete falsehoods^15,31^.

To investigate if attributions of intent affect whether people think a claim qualifies as true or false, we ran a series of experiments in which participants were shown claims of fact alongside the ground truth relevant to those claims. (In this paper, we use the term “ground truth” to refer to the true state of the world about which a claim is made.) Participants were tasked with judging the intentions of the information sources and reporting whether they would consider each claim to be true or false in light of the ground truth. By providing participants with the ground truth (which deviates from most research in the misinformation and belief-formation literatures), we ensure that participants know the precise accuracy of each provided claim. Thus, participants’ truth classifications—that is, whether they classify statements as true or false—should reflect not how much they believe a given claim, but whether they think a given claim qualifies as true or false.

Across all experiments, participants provided informed consent, protocols were approved by the Boston College Institutional Review Board, and procedures adhered to the guidelines and regulations of the Boston College Institutional Review Board.

## Study 1

Study 1 tests if and how the intentions people attribute to information sources predict whether people classify claims from those sources as true or false. To do so, we leveraged a context that has spurred contention in the misinformation era: reports about politically charged scientific findings.

### Methods

#### Participants

We recruited a nationally representative sample of U.S. Republicans and Democrats via Lucid (https://luc.id/). 877 participants completed our study, 205 of whom were excluded, per our preregistered exclusion criteria, for not completing the primary dependent measures, failing the attention check, and/or completing the study in less than 50% of the median completion time. Our final sample size was 672 (56.1% Democrat, 43.9% Republican; 55.2% female, 44.5% male, 0.3% non-binary/other; *M*_*age*_ = 51.51). See Supplementary Table S2 to view how the preregistered exclusions impacted the representativeness of the sample.

#### Materials and procedures

Participants saw six stimuli. Each stimulus contained a real scientific finding (e.g., “Each year between 4.7% and 13.2% of maternal deaths worldwide can be attributed to unsafe abortion”)^32^ and an ostensibly real (although actually fabricated) report from a news outlet about that scientific finding (e.g., “Abortion is killing mothers: up to 13.2% of maternal deaths can be attributed to abortion”). Supplying participants with the scientific finding on which the report was based ensured that all participants knew the ground truth of the topic the report detailed. Each report was crafted to be a logical consequence of the scientific finding, but to have a slant that supported the political agenda of either U.S. Republicans (as in the previous example) or U.S. Democrats. There were three Republican-slant stimuli and three Democrat-slant stimuli in total (See Supplementary Table S1 for the complete list of stimuli).

For each stimulus, the report was either attributed to “a news outlet” (i.e., the news outlet was not specified) or attributed to one of eight real news outlets: *CNN, Huffpost, MSNBC,* the *New York Times, Fox News, Breitbart,* the *Sean Hannity Show,* or the *Wall Street Journal*.

After reading each stimulus, participants first completed an intent-to-mislead measure and a truth-classification measure. The intent-to-mislead measure asked, “To what degree do you think the news outlet was trying to purposefully mislead its audience about the original scientific finding?” (1 = Not at all, 5 = A great deal). The truth-classification measure asked, “Assuming the original scientific finding is correct, would you consider the report from this news outlet to be true or false?” (1 = True, 0 = False). These two questions were presented in random order for each stimulus.

After the intent-to-mislead and truth-classification measures, participants completed the following five measures: (1) an accuracy measure asking, “Assuming the original scientific finding is correct, how accurate would you consider the report from the news outlet to be?” (1 = Not at all, 5 = A great deal); (2) a trust measure asking, “If this news outlet reported on a similar topic, how likely would you be to believe the report?” (1 = Extremely unlikely, 6 = Extremely likely); (3) an ethics-of-sharing measure asking, “How unethical do you think it would be for someone to share this news report on social media?” (1 = Not at all, 5 = A great deal); (4) a content-moderation measure asking, “If this news report were shared on social media, do you think the social media platform should take down the post?” (1 = Definitely not, 5 = Definitely); and (5) a belief measure asking, “Do you believe the original scientific finding?” (1 = Yes, 0 = Unsure, −1 = No). After completing the above measures for each of the six stimuli, participants answered three questions about each of the eight news outlets to which the news reports were randomly attributed: (1) an outlet-familiarity measure asking, “Before taking this survey, had you heard of the following news outlets?” (1 = Yes; 2 = No); (2) an outlet-consumption measure asking, “About how often do you consume news from the following outlets?” (1 = Not at all; 6 = Every day); and (3) an outlet political-leaning measure asking, “How politically conservative or politically liberal would you consider each of the following news outlets to be?” (1 = Extremely conservative; 7 = Extremely liberal).

We predicted participants would be more likely to classify a news report as false when they attributed greater intent-to-mislead to the news outlet.

### Results

To test the relationship between intent-to-mislead judgments and truth classifications, we assessed the repeated-measures correlation between participants’ intent-to-mislead ratings and their truth classifications among the full sample, as preregistered.

It is important to note that we also preregistered several other analyses, all of which are presented in the Supplementary Information. For these analyses, we expected that when the political leaning of the news outlet matched the political slant of the report, participants would attribute greater intent-to-mislead to the outlet and, in turn, be more likely to classify the report as false. Because of an error in the manipulation, the main-text analyses focus solely on the relationship between intent-to-mislead ratings and truth classifications. That said, preregistered analyses found the predicted effect of the manipulation on truth classifications among participants who passed the manipulation check (see Supplementary Fig. S2).

#### Correlation between intent-to-mislead judgments and truth classifications

**Figure 1:**
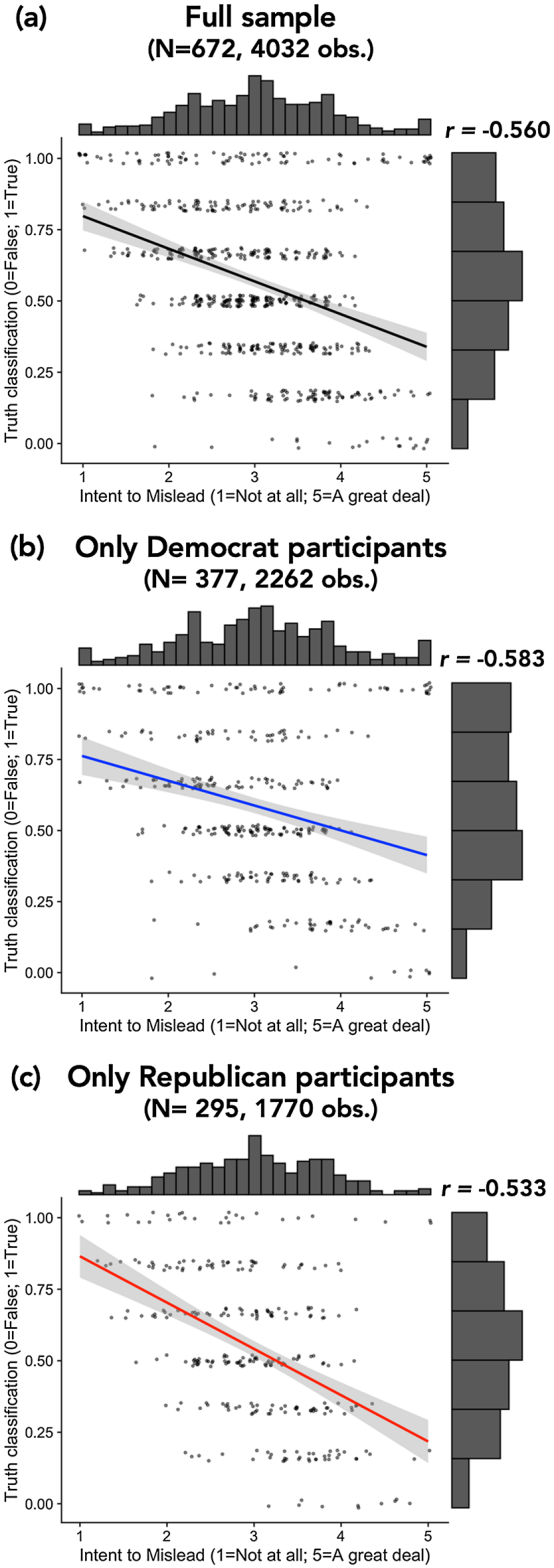
Relationship between intent-to-mislead judgments and truth classifications. The panels display scatterplots of intent-to-mislead judgments and truth classifications with the best-fit linear line among (**a**) the full sample, (**b**) only the Democrat participants, (**c**) only the Republican participants. Each individual data-point represents all observations for each participant averaged together (i.e., a person-level mean); histograms represent the distribution of values for the corresponding measure; *r* values represent the repeated-measures correlation between intent-to-mislead judgments and truth classifications; error bars around the best-fit line represent 95% confidence intervals; line color signifies political affiliation of the participants in the subsample (blue = Democrat; red = Republican; black = mixed).

Among the full sample, intent-to-mislead judgments were negatively associated with classifying a claim as true (*r* = −0.560, 95% CI [−0.583, −0.536], *p* < 0.001). In other words, the more participants judged news outlets as intending to mislead, the more likely participants were to classify the outlets’ reports as false (see Fig. 1). This relationship also emerged in every subsample of the data we assessed, including among only Democrats, only Republicans, only the observations for which participants reported that they believed the scientific finding, each news outlet, and each stimulus (see Supplementary Table S4).

### Discussion

Overall, we saw the expected relationship between intent-to-mislead judgments and truth classifications among the full sample: the more participants thought the news outlets were trying to mislead their audience, the less participants were likely to classify the reports as true. This result corroborates the findings from our pilot study (N = 132), which is reported in the Supplementary Information (see Study S1). Additionally, this relationship was robust across many subsamples of the data (see Supplementary Table S4). These results are only correlational, so they should be interpreted cautiously.

## Study 2

Compared to Study 1, Study 2 employed a larger number of stimuli (24 instead of 6). Additionally, Study 2 directly manipulated deceptive intent by explicitly stating the intentions of the information source (which we will refer to as the “deceive-inform intent manipulation”). Study 2 also varied whether the numerical information provided by the information source was a rounded number or a non-rounded number (which we will refer to as the “specific-approximate report manipulation”). This manipulation was designed to lead participants to infer that the source was intending to provide either an approximate account or a specific account of the true state.

### Methods

#### Participants

600 U.S. participants were recruited on Amazon Mechanical Turk via the CloudResearch-Approved Participants List^33^. Per our preregistered exclusion criteria, 91 participants were excluded for failing one or both attention checks, not completing all dependent measures, taking the study more than once, and/or completing the study in less than 50% of the median completion time. Our final sample size was 509 (55.0% female, 44.0% male, 0.8% non-binary/other, 0.2% undisclosed; *M*_*age*_ = 40.43).

#### Materials and procedures

All participants read 24 stimuli in a randomized order. Each stimulus described a state of the world using a numerical value, an information source making an assertion about that state of the world, and a description of the intention of that information source. For example, one stimulus read, “A journalist learned that 463 people attended a local politician’s campaign rally. The journalist, trying to [accurately inform the public / inflate the politician's popularity], reported that [499 / 500] people attended the rally,” where the sections in brackets indicate the information that was manipulated.

Each stimulus had a 2 (Inform Condition versus Deceive Condition) × 2 (Approximate Condition versus Specific Condition) structure. In the Inform Condition, the information source was said to be trying to accurately inform their audience (e.g., “trying to accurately inform the public”), while in the Deceive Condition the information source was said to be trying to deceive their audience (e.g., “trying to inflate the politician's popularity”). Meanwhile, in the Approximate Condition the report was manipulated to be a rounded number (e.g., “500”), while in the Specific Condition the report was manipulated to be a non-rounded number that was closer to the ground truth than the approximate number (e.g., “499”).

For each participant, 6 of the 24 stimuli were randomly selected to be in each of the four condition combinations. In contrast to Study 1, in which all of the reports participants classified were technically true given the supplied ground truth, all of the reports in Study 2 were technically false. All 24 stimuli are displayed in Supplementary Table S5.

After each stimulus, participants responded to four measures. The truth-classification measure asked, “Would you consider the information reported by [*information source*] to be true or false?” (1 = True, 0 = False). The trust measure asked, “How much would you trust other information provided by [*information source*]?” (1 = Completely distrust, 6 = Completely trust). The intent-to-deceive measure asked, “How much do you think [*information source*] was trying to deceive [*audience*]?” (1 = Not at all, 5 = A great deal). The intent-to-approximate measure asked, “How much do you think [*information source*] was trying to report a rough approximation of [*topic*]?” (1 = Not at all, 5 = A great deal). The portions in brackets indicate the type of stimulus-specific information that was piped into the question. Participants answered the truth-classification measure and the trust measure in a random order before answering the intent-to-deceive measure, followed by the intent-to-approximate measure.

We predicted that, across participants and stimuli, information sources’ reports would be more likely to be classified as true in the Inform Condition versus the Deceive Condition and in the Approximate Condition versus the Specific Condition.

### Results

#### Manipulation checks

Due to space limitations, results for the two intent measures (i.e., manipulation checks) are reported in the Supplementary Information, but, in brief, the manipulations successfully affected their corresponding measure of intent: there were higher intent-to-deceive ratings in the Deceive Condition versus the Inform Condition and higher intent-to-approximate ratings in the Approximate Condition versus the Specific Condition.

#### Effect of manipulations

#### Truth classifications

As preregistered, to assess the effects of our manipulations on our primary dependent variable, we ran a generalized linear mixed effects model predicting truth classifications (1 = True; 0 = False) as a function of the deceive-inform intent manipulation (effect-coded: Inform Condition = 0.5; Deceive Condition = −0.5), the specific-approximate report manipulation (effect-coded: Approximate Condition = 0.5; Specific Condition = −0.5), and the interaction between the manipulations. Participant-level and stimuli-level random intercepts as well as participant-level and stimuli-level random slopes for the deceive-inform intent manipulation, the specific-approximate report manipulation, and the interaction between the manipulations were included as random effects.

**Figure 2:**
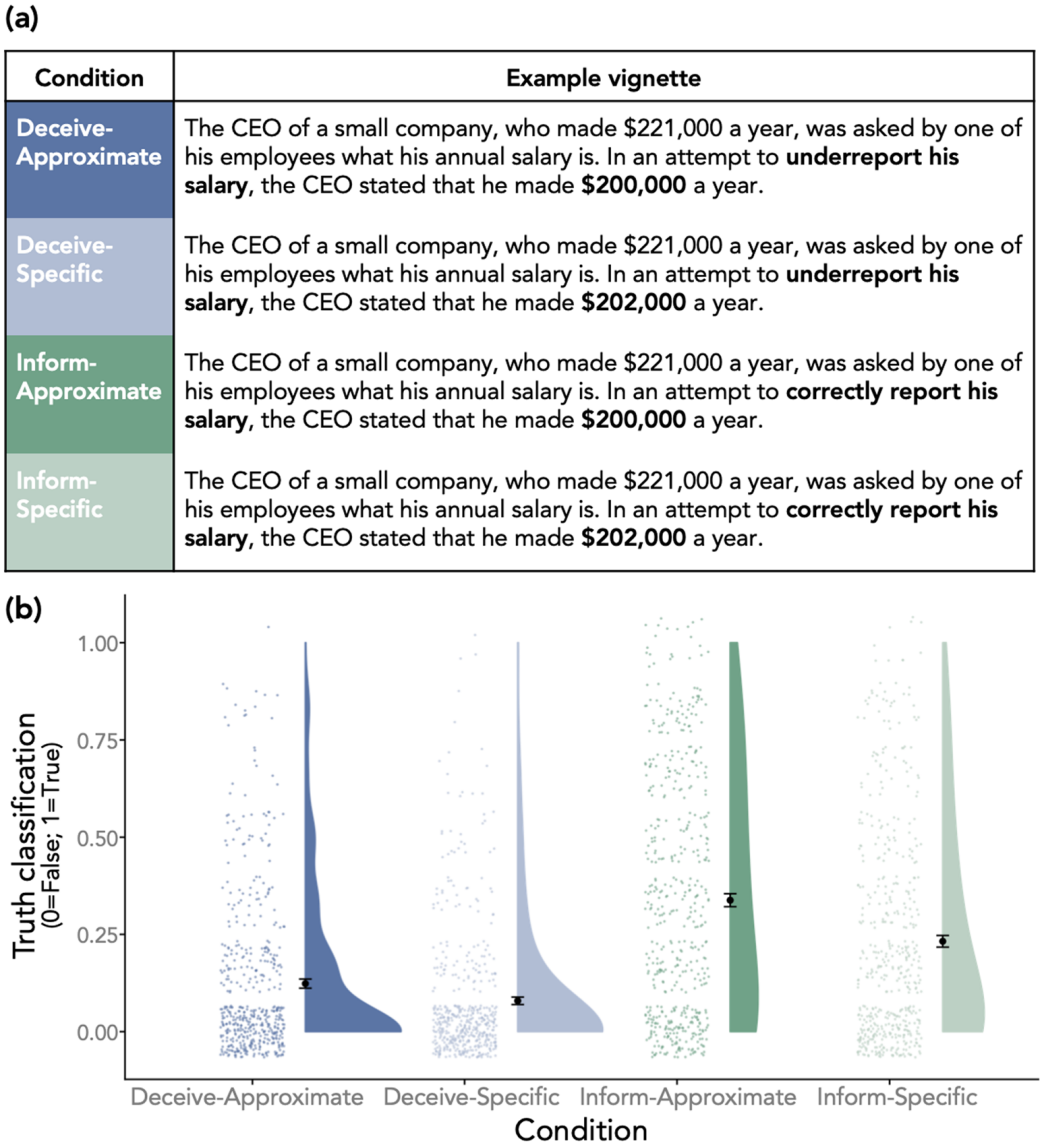
Example stimulus and results from Study 2. (**a**) One of the 24 stimuli shown as it appeared in each condition. The bolded text represents the information that was manipulated between conditions; it was not bolded for participants. (**b**) Truth classifications broken down by condition (full sample: N = 509, 12,216 observations). Each individual data-point represents all observations for each participant in the specified condition averaged together (i.e., a person-level condition mean); larger dots with error bars represent condition-level means and error bars represent 95% confidence intervals.

There was a significant effect of the deceive-inform intent manipulation, *b* = 2.57 (OR = 13.13, OR 95% CI [8.65–19.92]), *SE* = 0.21, *z* = 12.105, *p* < 0.001, such that reports were less likely to be classified as true in the Deceive Condition than in the Inform Condition, all else equal. There was also a significant effect of the specific-approximate report manipulation, *b* = 1.29 (OR = 3.62, OR 95% CI [2.42–5.42]), *SE* = 0.21, *z* = 6.26, *p* < 0.001, such that reports were more likely to be classified as true in the Approximate Condition than in the Specific Condition, all else equal. Finally, there was a significant (albeit, borderline-significant) interaction between the two manipulations, *b* = −0.73 (OR = 0.48, OR 95% CI [0.24–0.99]), *SE* = 0.37, *z* = −1.98, *p* = 0.047, such that the effect of the deceive-inform intent manipulation on truth classifications was stronger in the Approximate Condition compared to the Specific Condition. See Fig. 2, panel (b).

#### Robustness checks

We also conducted four robustness checks that tested whether the effects of the manipulations on truth classifications held when assessing: (1) only the first stimulus participants responded to (preregistered); (2) only the responses from participants for whom the manipulations influenced intent ratings in the expected directions (preregistered); (3) only the responses from participants who classified at least one stimulus as true and at least one as false (not preregistered); and (4) the number of participants who showed the predicted condition differences across their 24 responses (not preregistered). All four robustness checks corroborated the main effects of both manipulations observed in the primary analysis. However, robustness checks 1 and 3 did not find the significant interaction effect observed in the primary analysis and robustness check 4 found that only 19.3% of participants showed the full pattern of responses suggested by the primary-analysis interaction effect. Details for the robustness checks can be found in the Supplementary Information.

### Discussion

Study 2 tested 24 different stimuli in a within-subjects, repeated-measures design in which two features were manipulated: whether an information source was explicitly said to be trying to deceive or inform their audience and whether an information source reported a rounded or non-rounded number.

As predicted, participants’ truth classifications corresponded to the effects of the manipulations on intent ratings. Participants were more likely to judge an information source’s report as false when the information source was said to be trying to deceive their audience (Deceive Condition versus Inform Condition) as well as when the information source reported a non-rounded, incorrect value (Specific Condition versus Approximate Condition). These findings were robust to several different formulations of the data (see Supplementary Information).

Generally, the effects of each manipulation corroborate the findings from a between-subjects, single-stimulus version of this study (N = 744), which is reported in the supplement (see Study S3). Additionally, another study reported in the supplement (see Study S4) replicated the pattern of the Deceive Condition versus Inform Condition in a within-subjects, two-stimulus study (N = 880) in which the information source always reported a rounded number (equivalent to the Approximate Conditions in Study 2).

While we did observe a statistically significant interaction effect between the deceive-inform intent manipulation and the specific-approximate report manipulation, we recommend caution when interpreting it. The same interaction effect emerged in only one of the Study 2 robustness checks and did not emerge in Study S3.

Although the role of deceptive intent attributions in truth classifications remains our primary focus, incorporating the specific-approximate report manipulation and the intent-to-approximate measure in Study 2 made two important contributions. First, results from this manipulation suggest that the role of intent in truth classifications may not be limited to an intent to inform or deceive. Second, in line with Study 1, these results suggest that people make truth classifications based on the intentions they infer, not only based on intentions that are explicitly stated, such as those in the deceive-inform intent manipulation.

However, it is important to treat the results for the specific-approximate report manipulation more cautiously than the results for the deceive-inform intent manipulation because we did not find a statistically significant effect of the specific-approximate report manipulation in three preliminary studies reported in the supplement (see studies S2A–S2C). That said, we believe Study 2 is a much stronger test of the specific-approximate report manipulation than were studies S2A-S2C for three reasons: (1) Study 2 tested 24 stimuli, while studies S2A-S2C each only tested one stimulus; (2) Study 2 included many more participants per experimental cell (509 per cell) than even the largest of the three preliminary studies (about 197 per cell); (3) Study 2 collected multiple observations per participant, including observations in both the Approximate Condition and the Specific Condition. Moreover, another preliminary study (Study S3; N = 744) found significant effects of the specific-approximate report manipulation. Nevertheless, the stimuli in the two studies that found significant effects of the specific-approximate report manipulation (Study 2 and Study S3) included the deceive-inform intent manipulation, while the stimuli in the three studies that did not find significant effects of the specific-approximate report manipulation (Studies S2A-S2C) did not include the deceive-inform intent manipulation. So, we cannot rule out the possibility that the effect of the specific-approximate report manipulation only appears when the deceptive or informative intentions of the information source are made salient.

## General discussion

This paper tested whether attributions of intent affect what information people think qualifies as true. The two studies described above found that when participants attributed greater intent-to-deceive to an information source, they were more likely, in aggregate, to classify claims of fact from that information source as false. Study 1 found this relationship in the context of six news reports about scientific findings attributed to popular news outlets. Study 2 found this effect over 24 different scenarios in which the intent of the information source was explicitly stated. Study 2 also demonstrated that another type of attributed intent—the intent to approximate—predicted participants’ truth classifications. Participants were more likely to classify a claim as true when the information source provided an approximate account (i.e., a number that was incorrect, but rounded) rather than a precise account (i.e., a number that was incorrect and not rounded). Because participants in these studies always knew the ground truth, these results suggest that people do not only consider the objective accuracy of a claim when deciding whether the claim qualifies as true or false; rather, people additionally consider the information source’s intentions. In other words, our results suggest that the same exact claim might be considered true in one case and false in another, even when everyone involved knows precisely how accurate the claim is.

Why would attributions of intent affect what information people think qualifies as true? One possibility is that people use true–false labels not only to delineate fact from fiction, but also to signal the reliability of the information source. Labeling an information source’s claim as true or false not only signals to others the reliability of the claim in question, but also signals the reliability of the source. After all, a source whose claims are regularly called out for being false is unlikely to be considered a trustworthy source of information. Thus, when deciding whether a claim qualifies as true or false, people may account for the objective discrepancy between a claim and the ground truth as well as the perceived reliability of the source. As suggested by previous work in the person-perception literature^34^ and persuasion literature^35^, people might judge a source’s reliability as a function of the source’s intentions to help or harm (e.g., the intent to communicate accurate information) and the source’s ability to enact those intentions (e.g., the ability to communicate accurate information). The two manipulations in Study 2 arguably map well onto these two dimensions. The deceive-inform intent manipulation was designed to affect people’s perceptions of the source’s *intentions* to communicate accurate information. Meanwhile, the specific-approximate report manipulation might have affected people’s perceptions of the source’s *ability* to communicate accurate information. After all, when participants perceived the information source as trying to provide an exact account, they may have deemed the source as unable to communicate accurate information since that exact account was always wrong in our stimuli. Meanwhile, when participants perceived the information source as trying to provide an approximate account, they may have deemed the source as able to communicate accurate information since the approximate account reflected a reasonably rounded number in our stimuli. We hope future work tests this and other possible mechanisms directly.

A related question left open by our data is what specific features of information sources our measures of intent-to-mislead and intent-to-deceive are sensitive to. Recent work has distinguished people’s perceptions of bias (i.e., motivation to stake out a given position) and untrustworthiness (i.e., willingness to lie), arguing that these perceptions can differentially affect how people react to claims from information sources^36^. In our work, it is unclear whether people would be more likely to judge an information source as intending to mislead if they considered the source to be biased versus untrustworthy. That said, the stimuli in Study 1 were designed to contain no complete falsehoods, hinting that the intent-to-mislead measure in Study 1 may have picked up perceptions of bias primarily. In Study 2, on the other hand, the numbers reported by the information source in the Specific-Deceive Condition may have been interpreted as lies since the source was said to be attempting deception and the numbers were patently incorrect. Yet, perceptions of bias were likely still present in Study 2 because the information source often provided information that was selfishly beneficial (see Supplementary Table S5 for Study 2 stimuli). Future work should better disambiguate perceptions of source bias from untrustworthiness.

Regardless of the underlying psychological mechanisms at play, the fact that attributions of intent can affect what information people think qualifies as true—even when people know the ground truth—may shed light on disagreements in the post-truth era and contribute to the misinformation literature. As outlined in the introduction, the two dominant theoretical explanations in the misinformation literature suggest that either motivated reasoning^15^ or lack of critical reasoning^14^ are to blame for endorsement of misinformation. Both of these accounts focus on the role that discrepant beliefs about the true state of the world play in contemporary disagreements over the truth of claims of fact. Our work suggests that discrepant beliefs about the ground truth may not be necessary to produce these disagreements; discrepant attributions of intent may be sufficient. After all, in our studies, participants always knew the ground truth, ruling out discrepant beliefs about the ground truth as a likely explanation for our results. While our findings do not detract from the value of existing misinformation work, they reveal another avenue through which such disagreements can arise. This alternative avenue could suggest that interventions should focus on boosting trust in the intentions of important information sources, such as reputable journalistic, research, and governmental sources. On an optimistic note, our findings suggest that, by virtue of people’s tendency to label information from intentionally deceptive sources as false, people may be good watchdogs of intentionally misleading information. This could serve an important ecological niche in today’s information ecosystem given that claims that are not completely false can still be simultaneously misleading and convincing^15,31^.

Beyond this work’s relevance to the post-truth era, understanding what leads people to decide whether a piece of information qualifies as true or false is particularly important in light of recent large language models, such as ChatGPT and GPT-4. While these models have become immensely popular—ChatGPT is thought to be the fastest growing consumer app in history^37^—one critical limitation of large language models is that they do not always produce accurate content^38^. One popular technical approach to aligning the output of large language models with the goals of its users is called reinforcement learning from human feedback. As its name implies, this approach relies on feedback about the output of the language model from human users to train a reinforcement learning model that, in turn, helps the language model produce content that better matches human preferences^39^. Indeed, Google DeepMind recently used a similar approach to train their model GopherCite to provide accurate information more often^40^. As large language models become more pervasive and influential (e.g., being incorporated into online search engines, such as Bing), it is important to understand what lay people think qualifies as true since that same conceptualization of truth might be getting trained into these models through reinforcement learning from human feedback.

It is important to acknowledge that the studies in this paper had several limitations. First, although Studies 1, 2, S1, S2A, S2B, S2C, and S3 were preregistered, Study S4 was not preregistered because it was a small part of a large, exploratory study assessing how people think about truth along many different dimensions. Second, some of the preregistered analyses for Study 1 were not useful for testing the relationship between intent and truth because of an error in the manipulation (see Supplementary Information for more detail). Third, although the effect of the specific-approximate report manipulation was significant in Study 2 and Study S3, it was not significant in Studies S2A, S2B, or S2C, so the results for this manipulation should be interpreted cautiously (see Study 2 Discussion for more detail). Finally, it is possible that our use of within-subjects designs led to larger effects since participants had direct comparison points of other intentions that information sources might hold.

Additionally, there are at least two important constraints on the generality of our conclusions^41^. First, all of our participants were English-speaking, U.S. adults, and only Study 1 explicitly sought out a nationally representative sample. The terms “true” and “false” may well be conceptualized and/or employed differently in other languages and cultures. Second, we would not expect the influence of intent on truth classifications to emerge for just any pairing of claim and fact. We specifically chose claims that occupied the gray area between completely inaccurate and precisely accurate. Although we found a relationship between intent and truth classifications for claims that are logically true (Study 1) and claims that are logically false (Study 2), we would not expect intent to matter when claims are interpreted as either perfectly matching or drastically departing from the ground truth.

Nevertheless, we hope that the experimental paradigm employed in this paper, in which participants are supplied the ground truth, offers one way to directly explore how people think about the concept of truth. While there is a burgeoning literature on how people navigate misinformation^13–15^ and decades of research on how people judge truth when uncertain about the accuracy of a claim^12^, there is little work directly probing what information people think qualifies as true even when they know the objective accuracy of that information. We hope this paper brings the field one step closer to understanding how people conceptualize truth—a concept core to science, journalism, law, and so many other pillars of modern society.

## Supplementary Information

Supplementary Information.

## Data Availability

All preregistrations, materials, data, and code are openly available at https://osf.io/d3wa8.
