# Supplementary material for: The intentions of information sources can affect what information people think qualifies as true
Source: Sci Rep. 2023 May 12;13:7718. doi: 10.1038/s41598-023-34806-4 (PMC10182088; doi:10.1038/s41598-023-34806-4)
Supplement: Supplementary file 1 — Supplementary Information. [file 41598_2023_34806_MOESM1_ESM.docx]

**Supplementary Information for**

**“The intentions of information sources can affect what information people think qualifies as true”**

Isaac J. Handley-Miner^1*^, Michael Pope^2^, Richard Kenneth Atkins^2^, S. Mo Jones-Jang^3^, Daniel J. McKaughan^2^, Jonathan Phillips^4^, Liane Young^1^

^1^Department of Psychology and Neuroscience, Boston College, Chestnut Hill, MA 02467

^2^Department of Philosophy, Boston College, Chestnut Hill, MA 02467

^3^Department of Communication, Boston College, Chestnut Hill, MA 02467

^4^Program in Cognitive Science, Dartmouth College, Hanover, NH 03755

***Corresponding author**:

Isaac Handley-Miner

140 Commonwealth Ave, McGuinn 326, Chestnut Hill, MA 02467

518-265-3437

[isaac.handley-miner@bc.edu](mailto:isaac.handley-miner@bc.edu)

**Table of contents**

[**Links to preregistrations, data, materials, and code** 4](#_Toc133827582)

[**All data, code, materials, and copies of preregistrations:** 4](#_Toc133827583)

[**Original preregistrations:** 4](#_Toc133827584)

[**Additional Materials for Main Text Studies 1-3** 5](#_Toc133827585)

[**Study 1** 5](#_Toc133827586)

[***Stimuli*** 5](#_Toc133827587)

[***Sample demographics*** 6](#_Toc133827588)

[***Analyses not reported in main text*** 8](#_Toc133827589)

[**Primary preregistered analyses** 9](#_Toc133827590)

[**Follow-up analyses (non-preregistered)** 11](#_Toc133827591)

[**Secondary preregistered analyses** 14](#_Toc133827592)

[**Robustness checks (non-preregistered analyses)** 20](#_Toc133827593)

[**Reviewer-requested analyses (non-preregistered)** 21](#_Toc133827594)

[***Interpretation of analyses not reported in main text*** 22](#_Toc133827595)

[**Study 2** 23](#_Toc133827596)

[***Stimuli*** 23](#_Toc133827597)

[***Preregistered analyses not reported in main text*** 27](#_Toc133827598)

[**Robustness check 1: First-stimulus analysis** 27](#_Toc133827599)

[**Robustness check 2: Manipulation-success analysis** 30](#_Toc133827600)

[**Effect of condition on trust** 34](#_Toc133827601)

[**Correlation between truth and trust** 34](#_Toc133827602)

[***Non-preregistered analyses*** 35](#_Toc133827603)

[**Manipulation checks** 35](#_Toc133827604)

[**Robustness check 3: Variable true-false analysis** 35](#_Toc133827605)

[**Robustness check 4: Persons-as-effect-sizes analysis** 39](#_Toc133827606)

[**Correlations between truth and intent ratings** 41](#_Toc133827607)

[**Order of truth and trust measures** 42](#_Toc133827608)

[**Effect of discrepancy between claim and ground truth** 42](#_Toc133827609)

[**Supplementary Studies** 44](#_Toc133827610)

[**Study S1** 44](#_Toc133827611)

[***Methods*** 44](#_Toc133827612)

[**Participants** 44](#_Toc133827613)

[**Materials and procedures** 45](#_Toc133827614)

[***Results*** 47](#_Toc133827615)

[**Primary analyses (preregistered)** 47](#_Toc133827616)

[**Secondary analyses (preregistered)** 47](#_Toc133827617)

[**Exploratory analyses (not preregistered)** 49](#_Toc133827618)

[**Study S2A** 51](#_Toc133827619)

[***Methods*** 51](#_Toc133827620)

[**Participants** 51](#_Toc133827621)

[**Materials and procedures** 52](#_Toc133827622)

[***Results*** 52](#_Toc133827623)

[**Preregistered analyses** 52](#_Toc133827624)

[**Study S2B** 53](#_Toc133827625)

[***Methods*** 53](#_Toc133827626)

[**Participants** 53](#_Toc133827627)

[**Materials and procedures** 53](#_Toc133827628)

[***Results*** 54](#_Toc133827629)

[**Preregistered analyses** 54](#_Toc133827630)

[**Study S2C** 55](#_Toc133827631)

[***Methods*** 55](#_Toc133827632)

[**Participants** 55](#_Toc133827633)

[**Materials and procedures** 55](#_Toc133827634)

[***Results*** 56](#_Toc133827635)

[**Preregistered analyses** 56](#_Toc133827636)

[**Study S3** 57](#_Toc133827637)

[***Methods*** 57](#_Toc133827638)

[**Participants** 57](#_Toc133827639)

[**Materials and procedures** 57](#_Toc133827640)

[***Results*** 58](#_Toc133827641)

[**Primary analyses (preregistered)** 58](#_Toc133827642)

[**Secondary analyses (preregistered)** 59](#_Toc133827643)

[**Study S4** 62](#_Toc133827644)

[***Methods*** 62](#_Toc133827645)

[**Participants** 62](#_Toc133827646)

[**Materials and procedures** 62](#_Toc133827647)

[***Results*** 64](#_Toc133827648)

[***Discussion*** 65](#_Toc133827649)

[**References** 67](#_Toc133827650)

# **Links to preregistrations, data, materials, and code**

## **All data, code, materials, and copies of preregistrations:**

<https://osf.io/d3wa8/?view_only=be0a929b2f154a71bae6ce2d3a7836bb>

## **Original preregistrations:**

Study 1 preregistration:

<https://osf.io/kj4eq/?view_only=ff25a1e767b842bea663ed725c9ebec4>

Study 2 preregistration:

<https://osf.io/2gwn5/?view_only=fb05af9c974145d8b890ac7fff118d8b>

Study S1 preregistration:

<https://osf.io/gxp7f/?view_only=1b3d0c9ccdf4487b98bc6755553e0a03>

Study S2A preregistration:

<https://osf.io/rm7wp/?view_only=8f22720abb864bc79a2c46f5a30768da>

Study S2B preregistration:

<https://osf.io/u9x8q/?view_only=93050f0d6a7f457681e0c2280816e214>

Study S2C preregistration:

<https://osf.io/83rva/?view_only=4b33daa6d9f14b3a9861935a99e413ee>

Study S3 preregistration:

<https://osf.io/rmvb5/?view_only=eefa871f8f2147ceabd132cb59070eb4>

Study S4 preregistration:

[not preregistered]

# **Additional Materials for Main Text Studies 1-3**

## **Study 1**

### ***Stimuli***

**Table S1. Study 1 stimuli**

| **Stimulus Number** | **Stimulus** | **Political Slant** |
| --- | --- | --- |
| **1** | **Original scientific finding**: It is extremely likely that more than half of the observed increase in global average surface temperature from 1951 to 2010 was caused by humans.    **News report about scientific finding**: [{Republican-leaning outlet} / {Democrat-leaning outlet} / {unspecified}] reported, “Scientists can’t say for certain whether the increase in global surface temperature over the last several decades was caused by humans.” | Republican |
| **2** | **Original scientific finding**: Out of 2 million study participants who received at least one dose of a COVID-19 vaccine, 57 developed temporary heart complications.    **News report about scientific finding**: [{Republican-leaning outlet} / {Democrat-leaning outlet} / {unspecified}] reported, “In one study, dozens of people developed heart complications after receiving the COVID-19 vaccine. Some of those were after just one dose.” | Republican |
| **3** | **Original scientific finding**: Each year between 4.7% – 13.2% of maternal deaths worldwide can be attributed to unsafe abortion.    **News report about scientific finding**: [{Republican-leaning outlet} / {Democrat-leaning outlet} / {unspecified}] reported, “Abortion is killing mothers: up to 13.2% of maternal deaths can be attributed to abortion.” | Republican |
| **4** | **Original scientific finding**: Between August 31st and September 6th, 369 children in the U.S.—about 0.0005% of the U.S. youth population—were hospitalized per day, on average, with COVID-19.    **News report about scientific finding**: [{Republican-leaning outlet} / {Democrat-leaning outlet} / {unspecified}] reported, “There was a time this year when hundreds and hundreds of children were being hospitalized every single day due to COVID-19. Every single day.” | Democrat |
| **5** | **Original scientific finding**: There is about a 2 in 10 chance that human activity, such as carbon emissions, has affected tropical storm activity.    **News report about scientific finding**: [{Republican-leaning outlet} / {Democrat-leaning outlet} / {unspecified}] reported, “Human activity, such as carbon emissions, may have even influenced something as dangerous as tropical storm activity.” | Democrat |
| **6** | **Original scientific finding**: Nationwide, firearm purchasing and firearm violence increased substantially during the first months of the coronavirus pandemic. The magnitude of the increase in purchasing was not associated with the magnitude of the increase in firearm violence.    **News report about scientific finding**: [{Republican-leaning outlet} / {Democrat-leaning outlet} / {unspecified}] reported, “During the first few months of the COVID-19 pandemic, firearm purchasing increased. Do you know what else increased? Firearm violence.” | Democrat |

The bracketed text indicates the information that was manipulated depending on the condition in which participants saw each stimulus. “{Republican-leaning outlet}” was randomly auto-filled with one of four news outlets: Fox News, Wall Street Journal, Breitbart, or Sean Hannity Show. “{Democrat-leaning outlet}” was randomly auto-filled with one of four news outlets: MSNBC, New York Times, Huffpost, or CNN. “{unspecified}” was auto-filled with the text “A news outlet”. For each stimulus, participants saw the bolded labels of “Original scientific finding” and “News report about scientific finding,” as depicted in the second column. The political slant of the news report is indicated in the third column.

### ***Sample demographics***

As described in the main text, we recruited a nationally representative sample of U.S. Democrats and U.S. Republicans via Lucid (https://luc.id/). The demographics of the recruited sample (pre-exclusion sample) and the sample on which we ran analyses (post-exclusion sample) are reported in Table S2 below. As preregistered, participants were excluded for not completing the primary dependent measures, failing the attention check, and/or completing the study in less than 50% of the median completion time.

**Table S2. Demographic breakdown of participants in the pre-exclusion-criteria sample and post-exclusion-criteria sample**

|  | **Pre-exclusion sample (N=877)** | **Post-exclusion sample (N=672)** |
| --- | --- | --- |
| **Age** |  |  |
| 18-24 | 57 (6.5%) | 29 (4.3%) |
| 25-34 | 160 (18.2%) | 92 (13.7%) |
| 35-44 | 169 (19.3%) | 129 (19.2%) |
| 45-54 | 140 (16.0%) | 112 (16.7%) |
| 55-64 | 147 (16.8%) | 121 (18.0%) |
| 65+ | 201 (22.9%) | 186 (27.7%) |
| **Gender** |  |  |
| Male | 402 (45.8%) | 299 (​​44.5%) |
| Female | 473 (53.9%) | 371 (55.2%) |
| Non-binary/Other | 2 (0.2%) | 2 (0.3%) |
| **Race** |  |  |
| American Indian or Alaskan Native | 8 (0.9%) | 6 (0.9%) |
| Asian | 29 (3.3%) | 22 (3.3%) |
| Black or African American | 115 (13.1%) | 73 (10.9%) |
| Hispanic/Latino/a/x | 42 (4.8%) | 27 (4.0%) |
| Multiracial/Multiethnic | 43 (4.9%) | 37 (5.5%) |
| Native Hawaiian or Other Pacific Islander | 3 (0.3%) | 2 (0.3%) |
| White | 633 (72.2%) | 501 (74.6%) |
| Other | 4 (0.5%) | 4 (0.6%) |
| **Educational attainment** |  |  |
| Less than a high school diploma | 15 (1.7%) | 10 (1.5%) |
| High school degree or equivalent | 206 (23.5%) | 138 (20.5%) |
| Some college, no degree | 200 (22.8%) | 168 (25.0%) |
| Associate Degree | 102 (11.6%) | 80 (11.9%) |
| Bachelor's Degree | 228 (26.0%) | 175 (26.0%) |
| Postgraduate Degree | 125 (14.3%) | 100 (14.9%) |
| Not disclosed | 1 (0.1%) | 1 (0.1%) |
| **Political party** |  |  |
| Republican | 381 (43.4%) | 295 (43.9%) |
| Democrat | 495 (56.4%) | 377 (56.1%) |
| Not disclosed | 1 (0.1%) | 0 (0.0%) |

### ***Analyses not reported in main text***

As described in the main text, we expected that when the political leaning of the news outlet matched the political slant of the report, participants would attribute greater intent-to-mislead to the outlet and, in turn, be more likely to classify the report as false.

With respect to this hypothesis, Study 1 can be conceptualized as having three different conditions: (1) the Aligned Condition, in which the news report was attributed to a news outlet whose political leaning *aligned* with the political slant of the report (i.e., the outlet and the report were both Democrat-leaning or both Republican-leaning); (2) the Misaligned Condition, in which the news report was attributed to a news outlet whose political leaning *misaligned* with the political slant of the report (i.e., the outlet was Democrat-leaning and the report was Republican-leaning or vice-versa); (3) the Control Condition, in which the identity of the news outlet was not specified. We predicted that participants would attribute greater intent-to-mislead to the news outlets in the Aligned Condition compared to the Misaligned Condition. In turn, we expected that participants would be more likely to classify the report as false in the Aligned Condition compared to the Misaligned Condition.

Given these conditions, we intended to choose four outlets that more U.S. Democrats trust versus distrust and more U.S. Republicans distrust versus trust (“Democrat-leaning outlets”) and four outlets that more U.S. Republicans trust versus distrust and more U.S. Democrats distrust versus trust (“Republican-leaning outlets”). Using data from the Pew Research Center on trust in news outlets, *CNN*, *Huffpost*, *MSNBC*, and the *New York Times* met our criteria for Democrat-leaning outlets and *Fox News*, *Breitbart*, and the *Sean Hannity Show* met our criteria for Republican-leaning outlets (Pew Research Center, 2020). In error, we also classified the *Wall Street Journal* as meeting the criteria for Republican-leaning outlets; we discovered after collecting our data that more people trust versus distrust the Wall Street Journal among both Republicans and Democrats (Pew Research Center, 2020). This error is also reflected in the data: In response to the question, “How politically conservative or politically liberal would you consider each of the following news outlets to be?” (1 = Extremely conservative, 2 = Conservative, 3 = Slightly Conservative, 4 = Moderate, 5 = Slightly Liberal, 6 = Liberal, 7 = Extremely liberal), participants rated the *Wall Street Journal* as a 4.12 on average (see Figure S1 below). In other words, the average rating for the *Wall Street Journal* was closest to “moderate.” Given this error, we conducted a post-hoc set of analyses on a sample that contains no observations for which the *Wall Street Journal* was the alleged news outlet.

#### **Primary preregistered analyses**

Given a manipulation failure in our pilot study (see Study S1), we preregistered that we would run the models described below on both the full sample and the sample of those who passed the manipulation check (the “manipulation-success sample”). As preregistered, each participant was included in the manipulation-success sample only if their mean intent-to-mislead judgments were lower for the stimuli they saw in the Misaligned Condition compared to those they saw in the Aligned Condition. Out of the 672 participants in our full sample, 292 were included in the manipulation-success sample.

***Intent-to-mislead judgments*.** As preregistered, we ran a linear mixed effects model predicting intent-to-mislead judgments with random intercepts for participants. The primary dependent variable was a dummy-coded variable for condition (with the Aligned Condition as the comparison group). The model also included participant political party (Democrat = 1; Republican = 0), and the interactions between participant political party and each of the dummy-coded condition variables as controls.

Among the full sample, contrary to our hypothesis, we did not observe a significant effect of either the Misaligned Condition compared to the Aligned Condition, *b* = -0.11, *SE* = 0.08, *t* = -1.45, *p* = 0.147, or the Control Condition compared to the Aligned Condition, *b* = 0.13, *SE* = 0.08, *t* = 1.74, *p* = 0.083. We also did not observe significant effects of participant political party, *b* = 0.08, *SE* = 0.09, *t* = 0.846, *p* = 0.398, the interaction between Condition (Misaligned versus Aligned) and participant political party, *b* = 0.03, *SE* = 0.10, *t* = 0.26, *p* = 0.794, or the interaction between Condition (Control versus Aligned) and participant political party, *b* = -0.13, *SE* = 0.10, *t* = -1.24, *p* = 0.217.

Among the manipulation success sample, as hypothesized, we observed significant effects of both the Misaligned Condition compared to the Aligned Condition, *b* = -1.21, *SE* = 0.11, *t* = -11.32, *p* < 0.001, and the Control Condition compared to the Aligned Condition, *b* = -0.37, *SE* = 0.11, *t* = -3.43, *p* = 0.001, such that intent-to-mislead judgments were higher in the Aligned Condition compared to each of the other two conditions, all else equal. We did not observe a significant effect of participant political party, *b* = 0.08, *SE* = 0.12, *t* = 0.71, *p* = 0.478, the interaction between Condition (Misaligned versus Aligned) and participant political party, *b* = -0.07, *SE* = 0.15, *t* = -0.47, p = 0.641, or the interaction between Condition (Control versus Aligned) and participant political party, *b* = -0.07, *SE* = 0.15, *t* = -0.50, *p* = 0.615.

***Truth classifications*.** As preregistered, we ran a generalized linear mixed effects model predicting truth classifications (True=1; False=0) with random intercepts for participants. The primary dependent variable was a dummy-coded variable for condition (with the Aligned Condition as the comparison group). The model also included participant political party (Democrat = 1; Republican = 0), and the interactions between participant political party and each of the dummy-coded condition variables as controls.

Among the full sample, contrary to our hypothesis but in line with the intent-to-mislead results, we did not observe a significant effect of either the Misaligned Condition compared to the Aligned Condition, *b* = 0.13 (OR = 1.14, OR 95% CI [0.89 – 1.46]), *SE* = 0.13, *z* = 1.03, *p* = 0.303, or the Control Condition compared to the Aligned Condition, *b* = -0.10 (OR = 0.91, OR 95% CI [0.71 – 1.16]), *SE* = 0.13, *z* = -0.77, *p* = 0.442. We also did not observe significant effects of participant political party, *b* = 0.24 (OR = 1.27, OR 95% CI [0.96 – 1.68]), *SE* = 0.14, *z* = 1.69, *p* = 0.092, the interaction between Condition (Misaligned versus Aligned) and participant political party, *b* = -0.14 (OR = 0.87, OR 95% CI [0.62 – 1.21]), *SE* = 0.17, *z* = -0.84, *p* = 0.399, or the interaction between Condition (Control versus Aligned) and participant political party, *b* = 0.06 (OR = 1.06, OR 95% CI [0.76 – 1.48]), *SE* = 0.17, *z* = 0.34, *p* = 0.732.

Among the manipulation success sample, as hypothesized, we observed a significant effect of the Misaligned Condition compared to the Aligned Condition, *b* = 1.05 (OR = 2.85, OR 95% CI [1.97 – 4.14]), *SE* = 0.19, *z* = 5.52, *p* < 0.001, such that reports were classified as true less often in the Aligned Condition than the Misaligned Condition, all else equal. We did not observe a significant effect of the Control Condition compared to the Aligned Condition, *b* = 0.27 (OR = 1.31, OR 95% CI [0.91 – 1.87]), *SE* = 0.18, *z* = 1.47, *p* = 0.143. We also did not observe significant effects of participant political party, *b* = -0.06 (OR = 0.94, OR 95% CI [0.63 – 1.40]), *SE* = 0.20, *z* = -0.31, *p* = 0.756, the interaction between Condition (Misaligned versus Aligned) and participant political party, *b* = 0.10 (OR = 1.10, OR 95% CI [0.66 – 1.84]), *SE* = 0.26, *z* = 0.38, *p* = 0.702, or the interaction between Condition (Control versus Aligned) and participant political party, *b* = 0.34 (OR = 1.40, OR 95% CI [0.85 – 2.30]), *SE* = 0.25, *z* = 1.33, *p* = 0.182.

***Accuracy Judgments.*** As preregistered, we ran a linear mixed effects model predicting accuracy judgments with random intercepts for participants. The primary dependent variable was a dummy-coded variable for condition (with the Aligned Condition as the comparison group). The model also included participant political party (Democrat = 1; Republican = 0) and the interactions between participant political party and each of the dummy-coded condition variables as controls.

Among the full sample, contrary to our hypothesis but in line with the intent-to-mislead results, we did not observe a significant effect of either the Misaligned Condition compared to the Aligned Condition, *b =* 0.05*, SE =* 0.07*, t =* 0.68*, p* = 0.496 or the Control Condition compared to the Aligned Condition, *b =* -0.02*, SE =* 0.07*, t =* -0.22*, p* = 0.823. Participant political party was statistically significant, *b =* 0.24*, SE =* 0.08*, t =* 2.87*, p* = 0.004, such that Democrats reported higher accuracy judgments, all else equal. We did not observe a significant effect of the interaction between Control versus Aligned Condition and participant political party, *b =* -0.06*, SE =* 0.09*, t =* -0.66*, p* = 0.507, or the interaction between Misaligned versus Aligned Condition and participant political party, *b =* -0.09*, SE =* 0.09*, t =* -0.95*, p* = 0.341.

Among the manipulation success sample, as hypothesized, we observed significant effects of both the Control Condition compared to the Aligned Condition, *b =* 0.22*, SE =* 0*.*10*, t =* 2.215*, p* = 0.027, and the Misaligned Condition compared to the Aligned Condition, *b =* 0.62*, SE =* 0*.*10*, t =* 6.21*, p* < 0.001, such that accuracy judgments were lower in the Aligned Condition compared to each of the other two conditions, all else equal. We did not observe significant effects of participant political party, *b =* 0.01*, SE =* 0*.*11*, t =* 0.10*, p* = 0.919, the interaction between Control versus Aligned Condition and participant political party, *b =* 0.07*, SE =* 0.14*, t =* 0.55*, p* = 0.585, or the interaction between Misaligned versus Aligned Condition and participant political party, *b =* 0*.*06*, SE =* 0.14*, t =* 0.45*, p* = 0.654.

#### **Follow-up analyses (non-preregistered)**

As described above, we mistakenly classified the *Wall Street Journal* as meeting our criteria for a Republican-leaning outlet. Given this error, we first assessed how participants rated the political leanings of the eight news outlets to see whether the *Wall Street Journal* was rated as being Republican-leaning. Next, we ran our primary preregistered analyses (the same models as those described in the *primary preregistered analyses* section above) on the full sample, but without any observations for which the *Wall Street Journal* was the alleged news outlet. This led to 332 of the 4032 observations being dropped, leaving 3700 observations from 672 participants.

***Mean political leanings of outlets (full sample).*** We assessed how participants rated the political leanings of the eight news outlets by calculating for each outlet the mean value of the question, “How politically conservative or politically liberal would you consider each of the following news outlets to be?” (1 = Extremely conservative, 2 = Conservative, 3 = Slightly Conservative, 4 = Moderate, 5 = Slightly Liberal, 6 = Liberal, 7 = Extremely liberal). These values, along with their distributions and individual datapoints are presented in Figure S1 below.


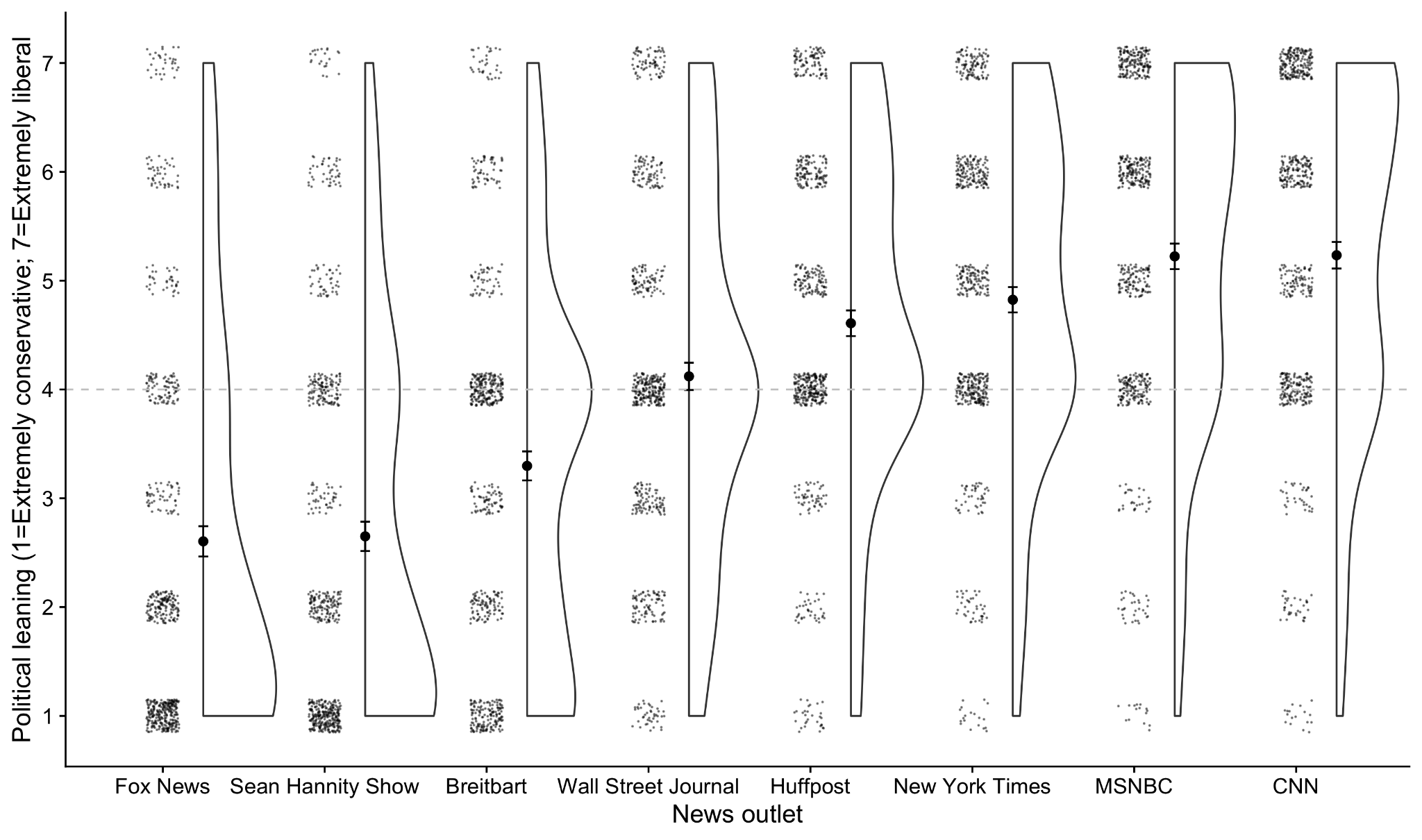


**Fig. S1.** Study 1 political leaning ratings of each news outlet. Each individual datapoint represents one rating from one participant for the specified news outlet; larger dots with error bars represent outlet-level means; error bars represent 95% confidence intervals; the dashed horizontal line represents the midpoint of the scale, which corresponds to the response “moderate.”

***Intent-to-mislead judgments (Wall Street Journal observations removed).*** Among the sample with *Wall Street Journal* observations removed, we observed a significant effect of the Misaligned Condition compared to the Aligned Condition, *b* = -0.21, *SE* = 0.08, *t* = -2.50, *p* = 0.013, such that intent-to-mislead judgments were higher in the Aligned Condition compared to the Misaligned Condition, all else equal. We did not observe a significant effect of the Control Condition compared to the Aligned Condition, *b* = 0.08, *SE* = 0.08, *t* = 1.02, *p* = 0.310. We also did not observe significant effects of participant political party, *b* = -0.00, *SE* = 0.09, *t* = -0.05, *p* = 0.959, the interaction between Condition (Misaligned versus Aligned) and participant political party, *b* = 0.19, *SE* = 0.11, *t* = 1.76, *p* = 0.078, or the interaction between Condition (Control versus Aligned) and participant political party, *b* = -0.05, *SE* = 0.11, *t* = -0.44, *p* = 0.658.

***Truth classifications (Wall Street Journal observations removed)*.** Among the sample with *Wall Street Journal* observations removed, we observed a significant effect of the Misaligned Condition compared to the Aligned Condition, *b* = 0.27 (OR = 1.32, OR 95% CI [1.01 – 1.72]), *SE* = 0.14, *z* = 2.00, *p* = 0.046, such that reports were classified as true less often in the Aligned Condition than the Misaligned Condition, all else equal. We did not observe a significant effect of the Control Condition compared to the Aligned Condition, *b* = -0.04 (OR = 0.96, OR 95% CI [0.74 – 1.24]), *SE* = 0.13, *z* = -0.31, *p* = 0.755. We observed a significant effect of participant political party, *b* = 0.33 (OR = 1.39, OR 95% CI [1.04 – 1.87]), *SE* = 0.15, *z* = 2.23, *p* = 0.026, such that reports were classified as true more often by Democrats, all else equal. We also observed a significant effect of the interaction between Condition (Misaligned versus Aligned) and participant political party, *b* = -0.37 (OR = 0.69, OR 95% CI [0.48 – 0.99]), *SE* = 0.18, *z* = -2.04, *p* = 0.042, such that the effect of political party on truth classifications was smaller in the Misaligned Condition relative to the Aligned Condition. Finally, we did not observe a significant effect of the interaction between Condition (Control versus Aligned) and participant political party, *b* = -0.04 (OR = 0.96, OR 95% CI [0.68 – 1.36]), *SE* = 0.18, *z* = -0.21, *p* = 0.837.

***Accuracy Judgments (Wall Street Journal observations removed).*** Among the sample with *Wall Street Journal* observations removed, we did not observe a significant effect of either the Misaligned Condition compared to the Aligned Condition, *b =* 0.13*, SE =* 0.07*, t =* 1.79*, p* = 0.074 or the Control Condition compared to the Aligned Condition, *b =* 0.04*, SE =* 0.07*, t =* 0.49*, p* = 0.622. Participant political party was statistically significant, *b =* 0.31*, SE =* 0.09*, t =* 3.55*, p* < 0.001, such that Democrats reported higher accuracy judgments, all else equal. The interaction between Misaligned versus Aligned Condition and participant political party was also significant, *b =* -0.23*, SE =* 0.10*, t =* -2.30*, p* = 0.022, such that the effect of political party on truth classifications was smaller in the Misaligned Condition relative to the Aligned Condition. The interaction between Control versus Aligned Condition and participant political party was not significant, *b =* -0.13*, SE =* 0.10*, t =* -1.38*, p* = 0.166.


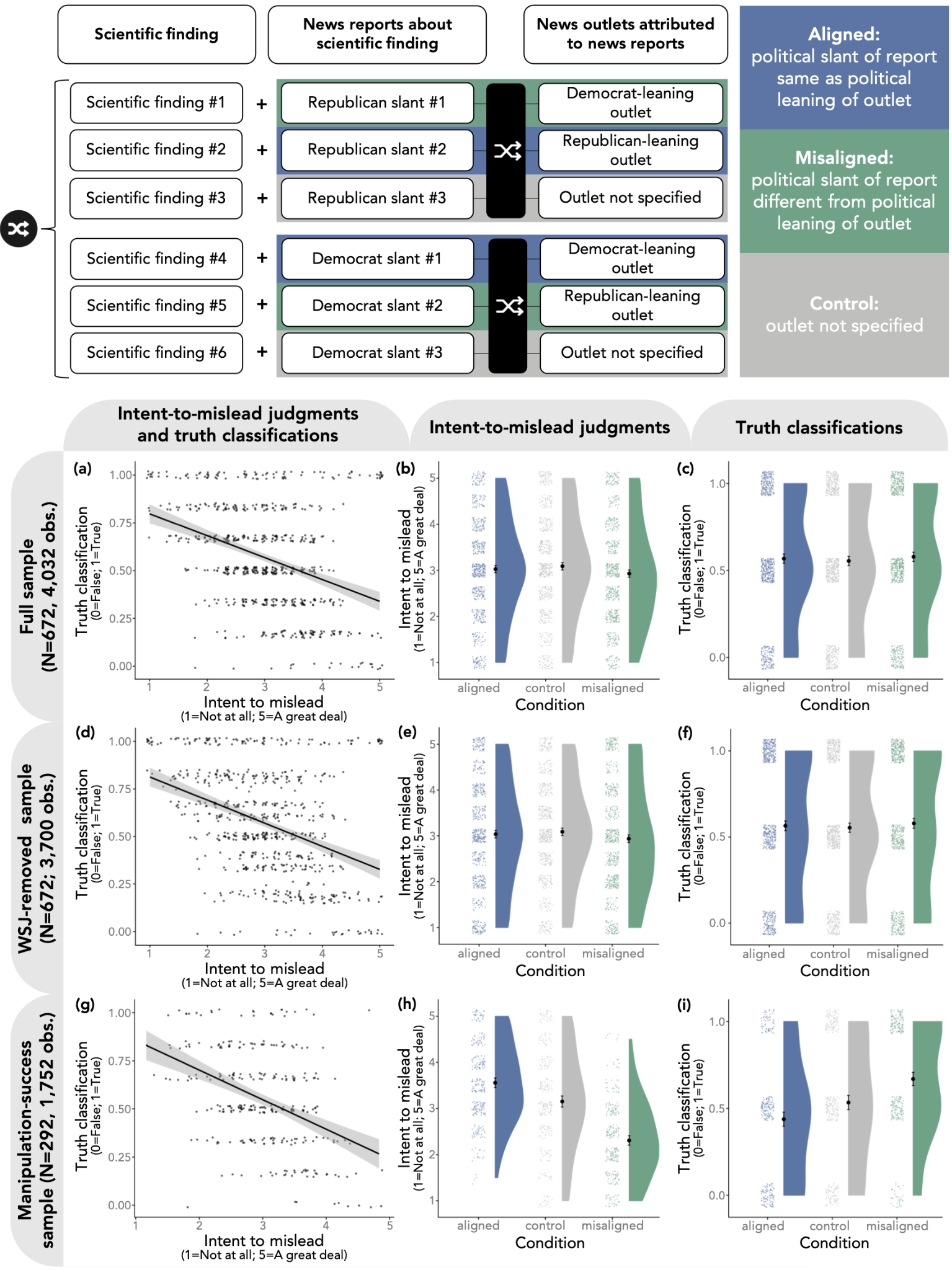


**Fig. S2.** Study 1 experimental design (top) and results (bottom). (a) Scatterplot between intent-to-mislead judgments and truth classifications with best-fit linear line, among the full sample (N=672, 4,032 observations); (b) Intent-to-mislead judgments, by condition, among the full sample (N=672, 4,032 observations); (c) Truth classifications, by condition, among the full sample (N=672, 4,032 observations); (d) Scatterplot between intent-to-mislead judgments and truth classifications with best-fit linear line, among the sample without *Wall Street Journal* observations (N=672, N=3,700); (e) Intent-to-mislead judgments, by condition, among the sample without *Wall Street Journal* observations (N=672, N=3,700); (f) Truth classifications, by condition, among the sample without *Wall Street Journal* observations (N=672, N=3,700); (g) Scatterplot between intent-to-mislead judgments and truth classifications with best-fit linear line, among the manipulation-success sample (N=292, 1,752 observations); (h) Intent-to-mislead judgments, by condition, among the manipulation-success sample (N=292, 1,752 observations); (i) Truth classifications, by condition, among the manipulation-success sample (N=292, 1,752 observations). In (a)-(i), each individual datapoint represents all observations for each participant in the specified condition averaged together (i.e., a person-level condition mean), larger dots with error bars represent condition-level means, and all error bars represent 95% confidence intervals.

#### **Secondary preregistered analyses**

We preregistered that we would run the following analyses, but we did not specify hypotheses for them.

***Correlation: Intent-to-mislead judgments and accuracy judgments (full sample).*** Within the full sample (N=672), intent-to-mislead judgments and accuracy judgments had a significant repeated-measures correlation in the full sample, *r =* -0.593, 95% CI [-0.614, -0.570], *p* < .001, such that greater intent-to-mislead judgments were associated with lower accuracy judgments.

***Correlation: Accuracy judgments and truth classifications (full sample).*** Within the full sample (N=672), accuracy judgments and truth classifications had a significant repeated-measures correlation in the full sample, *r =* 0.633, 95% CI [0.612, 0.653], *p* < .001, such that greater accuracy judgments were associated with a higher likelihood of a report being classified as true.

***2x3x2 analyses (truth; intent-to-mislead; accuracy).*** Another way of modeling the effects of our manipulations, while controlling for participants’ political affiliations is to treat this study as a 2x3x2 in which the political slant of the news report (Republican, Democrat), the political-leaning of the news outlet (Republican, Democrat, unspecified), and the political affiliation of the participant (Republican, Democrat) are each treated as a separate factor. We preregistered that we would also model the data from the full sample (N=672) by interacting all three of these factors in case this approach uncovered interactions between political party and either the slant of the news report or the leaning of the outlet.

As preregistered, using the three factors specified in the previous paragraph, we ran a generalized linear mixed effects model predicting truth classifications, a linear mixed effects model predicting intent-to-mislead judgments, and a linear mixed effects model predicting accuracy judgments. All three models included random intercepts for participants. The full output from these analyses can be seen in Tables S3a-S3c. These analyses were conducted on the dataset that included the *Wall Street Journal* observations, so should be interpreted cautiously.

**Table S3a. Study 1 model output from the generalized linear mixed effects model predicting truth classifications using the 2x3x2 approach**

|  | **Truth classifications** | | | | |
| --- | --- | --- | --- | --- | --- |
| *Predictors* | *log-Odds* | *std. Error* | *95% CI* | *z value* | *p* |
| (Intercept) | 0.29 | 0.14 | 0.01 – 0.56 | 2.07 | **0.039** |
| report slant | -0.38 | 0.18 | -0.73 – -0.02 | -2.09 | **0.037** |
| Outlet leaning  (Republican v none) | 0.27 | 0.18 | -0.09 – 0.63 | 1.47 | 0.141 |
| participant politics | -0.06 | 0.19 | -0.43 – 0.31 | -0.32 | 0.750 |
| Outlet leaning  (Democrat v none) | 0.23 | 0.18 | -0.12 – 0.59 | 1.29 | 0.198 |
| report slant * Outlet leaning (Republican v none) | -0.04 | 0.26 | -0.54 – 0.46 | -0.15 | 0.878 |
| report slant *  participant politics | 0.72 | 0.24 | 0.24 – 1.20 | 2.97 | **0.003** |
| Outlet leaning (Republican v none) * participant politics | -0.40 | 0.24 | -0.87 – 0.08 | -1.64 | 0.101 |
| report slant * Outlet leaning (Democrat v none) | -0.30 | 0.26 | -0.80 – 0.20 | -1.17 | 0.242 |
| participant politics * Outlet leaning (Democrat v none) | -0.09 | 0.24 | -0.57 – 0.38 | -0.38 | 0.708 |
| report slant * Outlet leaning (Republican v none) * participant politics | 0.08 | 0.34 | -0.60 – 0.75 | 0.22 | 0.827 |
| report slant * participant politics * Outlet leaning (Democrat v none) | 0.38 | 0.34 | -0.30 – 1.05 | 1.09 | 0.274 |
| **Random Effects** | | | | | |
| σ^2^ | 3.29 | | | | |
| τ_00_ _subjectid_ | 0.93 | | | | |
| ICC | 0.22 | | | | |
| N _subjectid_ | 672 | | | | |
| Observations | 4032 | | | | |
| Marginal R^2^ / Conditional R^2^ | 0.017 / 0.233 | | | | |

Model output from the generalized linear mixed effects model predicting truth classifications as a function of the political slant of the news report (Republican, Democrat), the political-leaning of the news outlet (Republican, Democrat, unspecified), the political affiliation of the participant (Republican, Democrat), and all 2-way and 3-way interactions, with random intercepts for participants. This analysis was preregistered and conducted on the full sample (N=672).

**Table S3b. Study 1 model output from the linear mixed effects model predicting intent-to-mislead judgments using the 2x3x2 approach**

|  | **Intent-to-mislead judgments** | | | | |
| --- | --- | --- | --- | --- | --- |
| *Predictors* | *Estimates* | *std. Error* | *95% CI* | *t value* | *p* |
| (Intercept) | 2.92 | 0.09 | 2.75 – 3.09 | 34.25 | **<0.001** |
| report slant | 0.39 | 0.11 | 0.18 – 0.60 | 3.63 | **<0.001** |
| Outlet leaning (Republican v none) | -0.29 | 0.11 | -0.50 – -0.08 | -2.68 | **0.007** |
| participant politics | 0.25 | 0.11 | 0.03 – 0.48 | 2.22 | **0.026** |
| Outlet leaning (Democrat v none) | -0.15 | 0.11 | -0.36 – 0.07 | -1.36 | 0.175 |
| report slant * Outlet leaning (Republican v none) | -0.06 | 0.15 | -0.36 – 0.24 | -0.38 | 0.705 |
| report slant * participant politics | -0.61 | 0.14 | -0.89 – -0.33 | -4.25 | **<0.001** |
| Outlet leaning (Republican v none) * participant politics | 0.48 | 0.14 | 0.20 – 0.77 | 3.37 | **0.001** |
| report slant * Outlet leaning (Democrat v none) | 0.17 | 0.15 | -0.13 – 0.46 | 1.09 | 0.275 |
| participant politics * Outlet leaning (Democrat v none) | -0.12 | 0.14 | -0.40 – 0.16 | -0.83 | 0.405 |
| report slant * Outlet leaning (Republican v none) * participant politics | -0.06 | 0.20 | -0.45 – 0.34 | -0.28 | 0.781 |
| report slant * participant politics *  Outlet leaning (Democrat v none) | -0.11 | 0.20 | -0.51 – 0.29 | -0.54 | 0.587 |
| **Random Effects** | | | | | |
| σ^2^ | 1.71 | | | | |
| τ_00_ _subjectid_ | 0.44 | | | | |
| ICC | 0.21 | | | | |
| N _subjectid_ | 672 | | | | |
| Observations | 4032 | | | | |
| Marginal R^2^ / Conditional R^2^ | 0.024 / 0.224 | | | | |

Model output from the linear mixed effects model predicting intent-to-mislead judgments as a function of the political slant of the news report (Republican, Democrat), the political-leaning of the news outlet (Republican, Democrat, unspecified), the political affiliation of the participant (Republican, Democrat), and all 2-way and 3-way interactions, with random intercepts for participants. This analysis was preregistered and conducted on the full sample (N=672).

**Table S3c. Study 1 model output from the linear mixed effects model predicting accuracy judgments using the 2x3x2 approach**

|  | **Accuracy judgments** | | | | |
| --- | --- | --- | --- | --- | --- |
| *Predictors* | *Estimates* | *std. Error* | *95% CI* | *t value* | *p* |
| (Intercept) | 2.67 | 0.08 | 2.52 – 2.83 | 34.17 | **<0.001** |
| report slant | -0.29 | 0.10 | -0.48 – -0.10 | -2.99 | **0.003** |
| Outlet leaning (Republican v none) | 0.14 | 0.10 | -0.06 – 0.33 | 1.39 | 0.164 |
| participant politics | -0.06 | 0.10 | -0.26 – 0.15 | -0.54 | 0.589 |
| Outlet leaning (Democrat v none) | -0.02 | 0.10 | -0.21 – 0.17 | -0.17 | 0.862 |
| report slant * Outlet leaning (Republican v none) | 0.01 | 0.14 | -0.26 – 0.28 | 0.05 | 0.961 |
| report slant * participant politics | 0.46 | 0.13 | 0.21 – 0.72 | 3.55 | **<0.001** |
| Outlet leaning (Republican v none) *  participant politics | -0.23 | 0.13 | -0.48 – 0.03 | -1.74 | 0.083 |
| report slant * Outlet leaning  (Democrat v none) | -0.09 | 0.14 | -0.36 – 0.18 | -0.64 | 0.522 |
| participant politics * Outlet leaning (Democrat v none) | 0.15 | 0.13 | -0.11 – 0.40 | 1.15 | 0.250 |
| report slant * Outlet leaning (Republican v none) * participant politics | 0.02 | 0.18 | -0.34 – 0.38 | 0.12 | 0.903 |
| report slant * participant politics *  Outlet leaning (Democrat v none) | 0.20 | 0.18 | -0.16 – 0.56 | 1.08 | 0.278 |
| **Random Effects** | | | | | |
| σ^2^ | 1.40 | | | | |
| τ_00_ _subjectid_ | 0.41 | | | | |
| ICC | 0.23 | | | | |
| N _subjectid_ | 672 | | | | |
| Observations | 4032 | | | | |
| Marginal R^2^ / Conditional R^2^ | 0.020 / 0.241 | | | | |

Model output from the linear mixed effects model predicting accuracy judgments as a function of the political slant of the news report (Republican, Democrat), the political-leaning of the news outlet (Republican, Democrat, unspecified), the political affiliation of the participant (Republican, Democrat), and all 2-way and 3-way interactions, with random intercepts for participants. This analysis was preregistered and conducted on the full sample (N=672).

#### **Robustness checks (non-preregistered analyses)**

To assess the robustness of the primary finding reported in the main text (i.e., a negative correlation between intent-to-mislead ratings and truth classifications), we tested the relationship between intent-to-mislead ratings and truth classifications across the following subsamples of the data: only Democrats, only Republicans, only the observations for which participants reported that they believed the scientific finding, only the manipulation-success sample, only the manipulation failure sample, each news outlet separately, and each stimulus separately. This relationship was tested using a repeated-measures correlation in all cases except when assessing the relationship among each news outlet separately and each stimulus separately. In those cases, Pearson's product moment correlations were used because there is only one observation per participant for each stimulus and each news outlet. All correlations are reported in Table S4 below.

**Table S4. Correlations between intent-to-mislead judgments and truth classifications among various subsamples**

| **Subsample of Study 1 data** | **Correlation coefficient** |
| --- | --- |
| Democrat participants | *r =* -0.583 |
| Republican participants | *r =* -0.533 |
| Observations for which participants reported that they believed the scientific finding | *r =* -0.597 |
| Manipulation-success sample | *r =* -0.580 |
| Manipulation-failure sample | *r =* -0.543 |
|  |  |
| *Each news outlet separately* |  |
| Breitbart | *r =* -0.512 |
| CNN | *r =* -0.483 |
| Fox News | *r =* -0.497 |
| Huffpost | *r =* -0.495 |
| MSNBC | *r =* -0.448 |
| New York Times | *r =* -0.482 |
| Sean Hannity Show | *r =* -0.466 |
| Wall Street Journal | *r =* -0.445 |
| Outlet not specified | *r* = -0.525 |
|  |  |
| *Each stimulus separately* |  |
| Stimulus #1 | *r =* -0.467 |
| Stimulus #2 | *r =* -0.446 |
| Stimulus #3 | *r =* -0.550 |
| Stimulus #4 | *r =* -0.535 |
| Stimulus #5 | *r =* -0.462 |
| Stimulus #6 | *r =* -0.472 |

#### **Reviewer-requested analyses (non-preregistered)**

***Effect of political leanings of outlets on intent-to-mislead judgments (Wall Street Journal observations removed).*** We assessed whether intent-to-mislead judgments were affected by whether a report was attributed to an outlet of the same political party as the participant or an outlet of the opposite political party as the participant. Because the average rating of the *Wall Street Journal’s* political leaning was very close to “moderate” (see above), we dropped the observations for which the *Wall Street Journal* was attributed to a report. Additionally, because we were interested in the effect of a same-party political leaning versus opposite-party political leaning, we dropped the observations for which no specific news outlet was attributed to the report. For the remaining 2,356 observations (from all 672 participants), we ran a linear mixed effects model predicting intent-to-mislead judgments as a function of whether the attributed news outlet had the same or opposite political leaning as the participant. The model included random intercepts and slopes for participants. There was a statistically significant effect, *b* = 0.37, *SE* = 0.06, *t* = 6.55, *p* < 0.001, such that participants reported higher average intent-to-mislead ratings for news outlets of the opposite political leaning compared to news outlets of the same political leaning. Given that the identity of the news outlets was randomly assigned to the reports, this finding could suggest that participants attributed an intent to mislead in a politically motivated fashion.

***Effect of measure order on relationship between intent-to-mislead and truth classifications.*** We assessed whether the order in which participants responded to the truth classification measure and the intent-to-mislead measure affected the relationship between participants’ intent-to-mislead judgments and their truth classifications. To test this, we ran a generalized linear mixed effects model predicting truth classifications (1 = true, 0 = false) as a function of intent-to-mislead judgments, measure order (truth first = 1, intent first = 0), and the interaction between intent-to-mislead judgments and measure order, with random intercepts for participants. We found a statistically significant effect of intent-to-mislead judgments, *b* = -1.01, *SE* = 0.05, *z* = -18.98, *p* < 0.001, such that greater attributions of intent-to-mislead was associated with a lower likelihood of classifying a claim as true. We also found a statistically significant effect of measure order, *b* = 1.00, *SE* = 0.23, *z* = 4.27, *p* < 0.001, such that participants were more likely to classify claims as true when they responded to the truth classification measure before the intent-to-mislead measure. Finally, we found a statistically significant interaction effect, *b* = -0.27, *SE* = 0.07, *z* = -3.92, *p* < 0.001, such that the negative relationship between intent-to-mislead and truth classifications was larger when participants responded to the truth classification measure before the intent-to-mislead measure. The observed interaction effect was in the opposite direction of what we expected. Because, as tested in Study 2 (and many of the supplemental studies), we anticipated intent-to-mislead to causally affect truth classifications, we expected that responding to the intent-to-mislead measure first would strengthen the observed relationship between the intent-to-mislead measure and truth classification measure.

To ensure that the relationship between intent-to-mislead ratings and truth classifications was still meaningful even when participants responded to the intent-to-mislead measure first, we then ran a repeated-measures correlation between intent-to-mislead ratings and truth classifications for only the observations in which participants responded to the intent-to-mislead measure first. Intent-to-mislead judgments were still negatively associated with classifying a claim as true (*r* = -0.500, 95% CI [-0.540, -0.458], *p* < .001), such that the more participants judged news outlets as intending to mislead, the more likely participants were to classify the outlets’ reports as false. This correlation of *r* = -0.500 was slightly lower than the correlation of *r* = -0.560 observed among the full sample (as suggested by the interaction effect in the previous analysis).

### ***Interpretation of analyses not reported in main text***

We expected that when the political leaning of the news outlet matched the political slant of the report (Aligned Condition), compared to when the political leaning of the outlet and the political slant of the report were at odds (Misaligned Condition), participants would attribute greater intent-to-mislead to the outlet and, in turn, be more likely to classify the report as false. Generally, the effects of the manipulation on truth classifications mirrored the effects of the manipulation on intent-to-mislead judgments. When the manipulation successfully affected intent-to-mislead ratings, it also affected truth classifications in the expected direction; when there was a null effect of the manipulation on intent-to-mislead ratings, there was also a null effect of the manipulation on truth classifications.

Among the full sample, including all of the observations that mistakenly treated the *Wall Street Journal* as a Republican-leaning outlet, we did not find evidence of the effect of the manipulation on truth classifications or intent-to-mislead ratings (preregistered analysis). When the *Wall Street Journal* observations were dropped, intent-to-mislead ratings were higher in the Aligned Condition compared to the Misaligned Condition, and participants classified the reports as false more often in the Aligned Condition compared to the Misaligned Condition (non-preregistered analysis). However, both of these effects had p-values between .01 and .05 and this analysis was not preregistered (since we of course did not anticipate the error of treating the *Wall Street Journal* as Republican-leaning), so they should be interpreted cautiously. Finally, among the participants for whom the manipulation successfully led to higher average intent-to-mislead ratings in the Aligned Condition compared to the Misaligned condition, the reports were classified as false more often in the Aligned Condition compared to the Misaligned Condition (preregistered analysis).

In aggregate, these results are in line with the correlations reported in the main text: when people attributed a greater intent to mislead to an information source, they were more likely to classify the source’s report as false.

## **Study 2**

### ***Stimuli***

**Table S5. Study 2 stimuli**

| **Stimulus Number** | **Stimulus** |
| --- | --- |
| 1 | A journalist learned that 463 people attended a local politician’s campaign rally. The journalist, trying to [accurately inform the public / inflate the politician's popularity], reported that [499 / 500] people attended the rally. |
| 2 | The CEO of a small company, who made $221,000 a year, was asked by one of his employees what his annual salary is. In an attempt to [correctly report his salary / underreport his salary], the CEO stated that he made [$202,000 / $200,000] a year. |
| 3 | A new social media company calculated that they had 91,040 active daily users on their platform. In a report to their shareholders, they wanted to [correctly convey the platform’s popularity / make the platform appear more successful than it was]. Their report stated that the platform had [97,290 / 100,000] active daily users. |
| 4 | A weathercaster for a local news channel learned that there was a 41% chance of a tornado in the area. With the goal of [communicating the actual severity of the storm / exaggerating the severity to gain more social media attention for the news channel], the weathercaster reported to viewers that there was a [48% / 50%] chance of a tornado. |
| 5 | An economist was asked to comment on the current state of the tech sector of the economy. Her research indicated that the tech sector was growing at a rate of 8.21% per year. Wanting to [help the public correctly understand how quickly this sector was growing / trick the public into investing in tech and boosting the value of her own investments], she reported that the tech sector was growing at [9.93% / 10%] per year. |
| 6 | In a recent survey conducted in a small city, a local polling organization found that 572 of the 1200 respondents thought the city’s mayor was doing a poor job. In an attempt to [accurately communicate the results / hurt the mayor’s political career by misrepresenting the results], the organization released a report stating that [591 / 600] of the 1200 respondents thought the city’s mayor was doing a poor job. |
| 7 | A team of scientists conducted a study on the side effects of an important new cancer medication and discovered that 11.3% of patients using this medication experienced side effects. In a TV interview, the scientists, trying to [accurately explain how safe the drug is / increase profits for the pharmaceutical company by misleading the public], reported that [10.3% / 10%] of patients experience side effects from this medication. |
| 8 | An auditor inspected a large company’s tax returns and discovered that the company had underreported their revenue by $10,433,219. The auditor, trying to [report the facts / help the company defraud the government] stated that the company had underreported their revenue by [$10,061,112 / $10,000,000]. |
| 9 | The prime minister of a small country reviewed the most recent jobs report, which showed that unemployment had decreased by 9.5% since she entered office. With the intention of [getting the facts right / exaggerating the numbers to make herself look better], the prime minister announced on TV that unemployment had decreased by [9.9% / 10%] during her term of office. |
| 10 | As part of his college applications, a high-school senior had to indicate how much community service he had completed during high school. He saw from the hours he had tracked that he completed 183 hours of community service. With the hopes of [accurately demonstrating his commitment to community service / deceiving the colleges into thinking he had completed more community service], he reported on the applications that he had completed [198 / 200] hours. |
| 11 | A researcher created a survey to administer to a group of respondents. During the first round of data collection, it took participants 37 minutes, on average, to complete the survey. For the second round of data collection, the researcher intended to [accurately inform respondents about the length of the survey / mislead respondents into thinking the survey was shorter]. The researcher told prospective respondents that the survey takes an average of [32 / 30] minutes to complete. |
| 12 | A used car salesperson was asked by a customer about the average lifetime of a particular SUV. The salesperson had read earlier that day that this SUV had an average lifetime of 189,500 miles. In an attempt to [get the facts right / make the car seem better than it is], the salesperson told the customer that the lifetime of this SUV was [197,250 / 200,000] miles. |
| 13 | An animal shelter was asked by an animal rights group how many animals they had housed the previous year. The shelter checked their records, and saw that they had housed 91 animals. Meaning to [truthfully relay the information / exaggerate the number in the hopes of receiving funding from the group], the shelter reported to the animal rights group that they had housed [98 / 100] animals the previous year. |
| 14 | While creating a documentary about an outbreak of a rare disease, the filmmaker learned that the small village featured in the documentary had recorded 912 cases of the disease. With the intention of [correctly communicating the facts / making the number of cases seem even more extreme], the filmmaker stated in the documentary that [991 / 1000] cases were recorded in the village. |
| 15 | A customer at a flower shop was interested in purchasing a large bouquet of roses. Before buying the roses, she asked the florist how many roses the bouquet contained. The florist had created the bouquet that morning with 26 roses. Trying to [accurately inform the customer / make the price seem better], the florist told the customer that it contained [29 / 30] roses. |
| 16 | An ophthalmology technician calculated that the average time it took her to complete an optical biometry was 23 minutes. When asked by upper management, the technician, intending to [accurately report how long it took her / appear more competent than she was], reported taking [21 / 20] minutes on average for each biometry. |
| 17 | A tech blogger wrote a review about a new model of headphones that retail for $219. In his review, he intended to [accurately report the price of the headphones / convince his readers to purchase through his affiliate program by misstating the actual price]. His review stated that the price of the headphones was [$204 / $200]. |
| 18 | In an interview with a journalist, a hospital manager was asked about the number of deaths the intensive care unit had experienced. The manager had checked the records before the interview and seen that 114 patients in the intensive care unit had died that year. In an attempt to [provide accurate numbers / downplay the number of deaths], the manager reported that [103 / 100] patients had died. |
| 19 | The mayor of a large city was informed that lead levels in the city water were at a dangerous level of 113 µg/L. When asked about the lead levels during a press conference, the mayor, trying to [accurately describe / downplay the danger of] the state of the city water, reported that the lead levels were [104 / 100] μg/L. |
| 20 | Two men were planning their wedding and shopping around for a venue. When they finally found a venue they liked, they asked the owner about the venue’s maximum capacity. The owner had set the maximum capacity at 119 people earlier that month. Attempting to [provide the couple with accurate information about the venue / dissuade the couple by misrepresenting the capacity], she told them that the maximum capacity was [102 / 100]. |
| 21 | A farmer requested a bank loan of $10,000 to purchase seed for the upcoming planting season. Based on the financial documents the farmer submitted, the bank’s loan officer calculated that the bank could approve a loan of $9,481. When meeting with the store owner, the loan officer attempted to [give the farmer correct figures about the loan calculation / mislead the farmer into seeking a loan elsewhere because he distrusted people without a college education], and told the farmer that the bank could offer a loan of [$9,017 / $9,000]. |
| 22 | A middle school teacher, prepping for his history class later that day, saw in the textbook that 143 members of the U.S. Military were killed in action during the Gulf War of 1991. During class, the teacher wanted to [report the accurate number of U.S. military deaths / downplay the death toll because he had been a supporter of the war]. He told his students that [113 / 100] members of the U.S. Military were killed. |
| 23 | A doctor learned about a new experimental treatment for leukemia from a pharmaceutical representative. The new treatment was estimated to be 4.3% more effective than existing treatments. The doctor intended to [be fully transparent with his patients about the efficacy of this treatment / mislead his patients into trying treatment in order to win favor with the attractive pharmaceutical representative]. He told his patients that the treatment was estimated to be [4.9% / 5%] more effective than existing treatments. |
| 24 | A waitress asked the owner of a restaurant how much tip money had come in the previous night. From her accounting that morning, the owner had calculated that there were $1,129 in tips. The owner, meaning to [accurately state the amount / underreport the number so she could keep the leftover tip money for herself], told the waitress that there were [$1,011 / $1,000] in tips. |

The 24 stimuli used in Study 2. The bracketed sections represent the information that was manipulated depending on the condition in which participants saw the stimulus. For the first set of manipulated information, in the Inform Condition, the information source was said to be trying to accurately inform their audience, while in the Deceive Condition the information source was said to be trying to deceive their audience. For the second set of manipulated information, in the Approximate Condition the report was a rounded number, while in the Specific Condition the report was a non-rounded number that was closer to the true state than the approximate number.


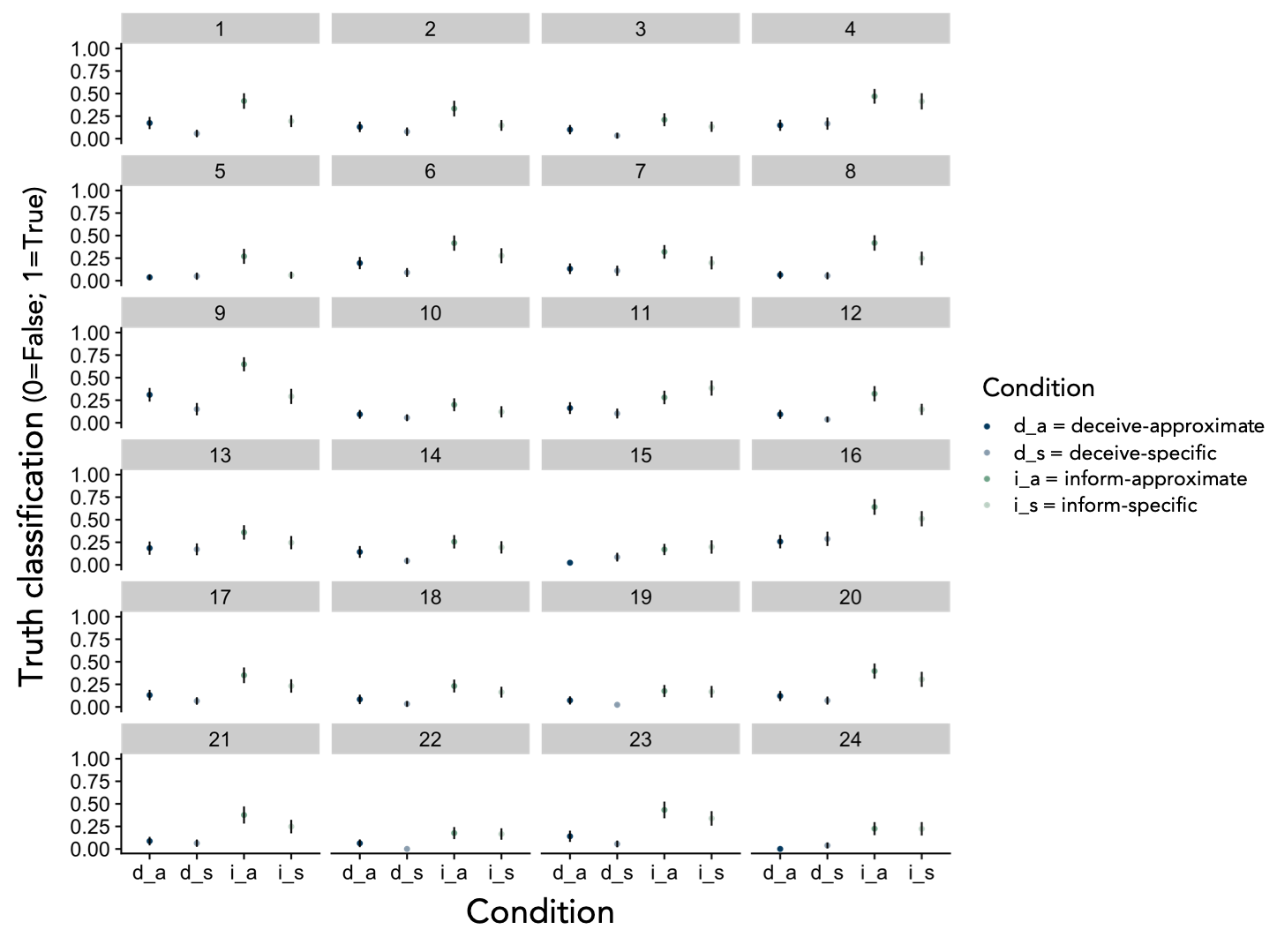


**Fig. S3.** Study 2 condition-level means faceted by stimulus. The number for each facet refers to the stimulus number indicated in Table S5 above.

### ***Preregistered analyses not reported in main text***

#### **Robustness check 1: First-stimulus analysis**

We preregistered that we would run analyses assessing only the first stimulus participants responded to (akin to a between-subjects design in which participants are nested within stimuli and condition) in case our findings were an artifact of the within-subjects, repeated-measures design.

***Truth.*** We ran a generalized linear mixed effects model predicting truth classifications as a function of the deceive-inform intent manipulation (effect-coded: Inform = 0.5; Deceive = -0.5), the specific-approximate report manipulation (effect-coded: Approximate = 0.5; Specific = -0.5), and the interaction between the manipulations. This model had the following random effects structure: stimuli-level random intercepts and stimuli-level random slopes for the deceive-inform intent manipulation, the specific-approximate report manipulation, and the interaction between the manipulations. Note that we mistakenly preregistered that this model would also include participant-level random intercepts—because there is only one observation per participant in this analysis, it does not make sense to model participant-level random effects. Results from this model can be seen in Table S6a.

**Table S6a. Study 2 model results from the generalized linear mixed effects model predicting truth classifications (first-stimulus analysis)**

|  | **Truth classifications** | | | | |
| --- | --- | --- | --- | --- | --- |
| *Predictors* | *Log-Odds* | *std. Error* | *95% CI* | *z value* | *p* |
| (Intercept) | -1.69 | 0.26 | -2.20 – -1.18 | -6.47 | **<0.001** |
| inform vs deceive | 1.18 | 0.36 | 0.48 – 1.89 | 3.29 | **0.001** |
| approximate vs specific | 0.74 | 0.33 | 0.08 – 1.39 | 2.21 | **0.027** |
| inform vs deceive *  approximate vs specific | 0.13 | 0.66 | -1.16 – 1.42 | 0.19 | 0.845 |
| **Random Effects** | | | | | |
| σ^2^ | | 3.29 |  |  |  |
| τ_00_ _stimulus_ |  | 0.91 |  |  |  |
| τ_11_ _stimulus.inform_vs_deceive_ |  | 0.20 |  |  |  |
| τ_11_ _stimulus.approximate_vs_specific_ |  | 0.02 |  |  |  |
| τ_11_ _stimulus.inform_vs_deceive:approximate_vs_specific_ | | 0.10 |  |  |  |
| ρ_01_ |  | -0.81 |  |  |  |
|  |  | 0.01 |  |  |  |
|  |  | 0.88 |  |  |  |
| N _stimulus_ |  | 24 |  |  |  |
| Observations |  | 509 |  |  |  |
| Marginal R^2^ / Conditional R^2^ |  | 0.129 / NA | |  |  |

***Trust.*** We ran a linear mixed effects model predicting trust as a function of the deceive-inform intent manipulation (effect-coded: Inform = 0.5; Deceive = -0.5), the specific-approximate report manipulation (effect-coded: Approximate = 0.5; Specific = -0.5), and the interaction between the manipulations. This model had the following random effects structure: stimuli-level random intercepts and stimuli-level random slopes for the deceive-inform intent manipulation, the specific-approximate report manipulation, and the interaction between the manipulations. Note that we mistakenly preregistered that this model would also include participant-level random intercepts—because there is only one observation per participant in this analysis, it does not make sense to model participant-level random effects. Results from this model can be seen in Table S6b.

**Table S6b. Study 2 model results from the linear mixed effects model predicting trust judgments (first-stimulus analysis)**

|  | **Trust judgments** | | | | |  |
| --- | --- | --- | --- | --- | --- | --- |
| *Predictors* | *Estimates* | *std. Error* | *95% CI* | *t value* | *p* |  |
| (Intercept) | 2.81 | 0.10 | 2.62 – 3.01 | 28.53 | **<0.001** |  |
| inform vs deceive | 1.01 | 0.13 | 0.75 – 1.26 | 7.82 | **<0.001** |  |
| approximate vs specific | 0.47 | 0.11 | 0.25 – 0.69 | 4.23 | **<0.001** |  |
| inform vs deceive *  approximate vs specific | -0.02 | 0.20 | -0.42 – 0.38 | -0.10 | 0.922 |  |
| **Random Effects** | | | | | |  |
| σ^2^ |  | 1.20 | | | | |
| τ_00_ _stimulus_ |  | 0.17 | | | | |
| τ_11_ _stimulus.inform_vs_deceive_ |  | 0.15 | | | | |
| τ_11_ _stimulus.approximate_vs_specific_ |  | 0.06 | | | | |
| τ_11_ _stimulus.inform_vs_deceive:approximate_vs_specific_ | | 0.06 | | | | |
| ρ_01_ |  | -0.11 | | | | |
|  |  | 0.87 | | | | |
|  |  | -0.34 | | | | |
| N _stimulus_ |  | 24 | | | | |
| Observations |  | 509 | | | | |
| Marginal R^2^ / Conditional R^2^ |  | 0.205 / NA | | | | |

#### **Robustness check 2: Manipulation-success analysis**

We preregistered that we would run analyses assessing the responses from only the participants who passed both of our manipulation checks. A participant was classified as passing the deceive-inform intent manipulation check if, across their responses to the 24 stimuli, their mean intent-to-deceive judgment in the Deceive Condition was greater than their mean intent-to-deceive judgment in the Inform Condition. A participant was classified as passing the specific-approximate report manipulation check if, across their responses to the 24 stimuli, their mean intent-to-approximate judgment in the Approximate Condition was greater than their mean intent-to-approximate judgment in the Specific condition. 365 of the 509 participants included in the main-text analyses passed both manipulation checks, and are included in the analyses below.

***Truth.*** We ran a generalized linear mixed effects model predicting truth classifications as a function of the deceive-inform intent manipulation (effect-coded: Inform = 0.5; Deceive = -0.5), the specific-approximate report manipulation (effect-coded: Approximate = 0.5; Specific = -0.5), and the interaction between the manipulations. This model had the following random effects structure: participant-level and stimuli-level random intercepts as well as participant-level and stimuli-level random slopes for the deceive-inform intent manipulation, the specific-approximate report manipulation, and the interaction between the manipulations. Results from this model can be seen in Table S7a.

**Table S7a. Study 2 model results from the generalized linear mixed effects model predicting truth classifications (manipulation-success analysis)**

|  | **Truth classifications** | | | | |  |
| --- | --- | --- | --- | --- | --- | --- |
| *Predictors* | *Log-Odds* | *std. Error* | *95% CI* | *z value* | *p* |  |
| (Intercept) | -3.15 | 0.26 | -3.67 – -2.63 | -11.94 | **<0.001** |  |
| inform vs deceive | 2.82 | 0.30 | 2.23 – 3.42 | 9.28 | **<0.001** |  |
| approximate vs specific | 1.76 | 0.29 | 1.18 – 2.33 | 6.01 | **<0.001** |  |
| inform vs deceive *  approximate vs specific | -1.11 | 0.56 | -2.21 – -0.01 | -1.99 | **0.047** |  |
| **Random Effects** | | | | | |  |
| σ^2^ |  | 3.29 | | | | |
| τ_00_ _subjectid_ |  | 4.32 | | | | |
| τ_00_ _stimulus_ |  | 0.91 | | | | |
| τ_11_ _subjectid.inform_vs_deceive_ |  | 1.63 | | | | |
| τ_11_ _subjectid.approximate_vs_specific_ |  | 0.45 | | | | |
| τ_11_ _subjectid.inform_vs_deceive:approximate_vs_specific_ | | 1.07 | | | | |
| τ_11_ _stimulus.inform_vs_deceive_ |  | 0.28 | | | | |
| τ_11_ _stimulus.approximate_vs_specific_ |  | 0.26 | | | | |
| τ_11_ _stimulus.inform_vs_deceive:approximate_vs_specific_ | | 0.38 | | | | |
| ρ_01_ |  | -0.23 | | | | |
|  |  | -0.56 | | | | |
|  |  | 0.68 | | | | |
|  |  | -0.47 | | | | |
|  |  | -0.44 | | | | |
|  |  | 0.88 | | | | |
| N _subjectid_ |  | 365 | | | | |
| N _stimulus_ |  | 24 | | | | |
| Observations |  | 8760 | | | | |
| Marginal R^2^ / Conditional R^2^ | | 0.463 / NA | | | | |

***Trust.*** We ran a linear mixed effects model predicting trust classifications as a function of the deceive-inform intent manipulation (effect-coded: Inform = 0.5; Deceive = -0.5), the specific-approximate report manipulation (effect-coded: Approximate = 0.5; Specific = -0.5), and the interaction between the manipulations. This model had the following random effects structure: participant-level and stimuli-level random intercepts as well as participant-level and stimuli-level random slopes for the deceive-inform intent manipulation, the specific-approximate report manipulation, and the interaction between the manipulations. Results from this model can be seen in Table S7b.

**Table S7b. Study 2 model results from the linear mixed effects model predicting trust judgments (manipulation-success analysis)**

|  | **Trust judgments** | | | | |  |
| --- | --- | --- | --- | --- | --- | --- |
| *Predictors* | *Estimates* | *std. Error* | *95% CI* | *t value* | *p* |  |
| (Intercept) | 2.82 | 0.08 | 2.66 – 2.99 | 33.56 | **<0.001** |  |
| inform vs deceive | 1.34 | 0.08 | 1.18 – 1.50 | 16.63 | **<0.001** |  |
| approximate vs specific | 0.31 | 0.05 | 0.22 – 0.40 | 6.65 | **<0.001** |  |
| inform vs deceive *  approximate vs specific | 0.11 | 0.06 | -0.02 – 0.23 | 1.65 | 0.099 |  |
| **Random Effects** | | | | | |  |
| σ^2^ |  | 0.80 | | | | |
| τ_00_ _subjectid_ |  | 0.28 | | | | |
| τ_00_ _stimulus_ |  | 0.15 | | | | |
| τ_11_ _subjectid.inform_vs_deceive_ |  | 0.49 | | | | |
| τ_11_ _subjectid.approximate_vs_specific_ |  | 0.06 | | | | |
| τ_11_ _subjectid.inform_vs_deceive:approximate_vs_specific_ | | 0.14 | | | | |
| τ_11_ _stimulus.inform_vs_deceive_ |  | 0.11 | | | | |
| τ_11_ _stimulus.approximate_vs_specific_ |  | 0.04 | | | | |
| τ_11_ _stimulus.inform_vs_deceive:approximate_vs_specific_ | | 0.05 | | | | |
| ρ_01_ |  | 0.20 | | | | |
|  |  | 0.03 | | | | |
|  |  | -0.38 | | | | |
|  |  | -0.25 | | | | |
|  |  | -0.13 | | | | |
|  |  | -0.28 | | | | |
| N _subjectid_ |  | 365 | | | | |
| N _stimulus_ |  | 24 | | | | |
| Observations |  | 8760 | | | | |
| Marginal R^2^ / Conditional R^2^ |  | 0.372 / NA | | | | |

#### **Effect of condition on trust**

We preregistered that we would assess the effects of our manipulations on the trust measure by running a linear mixed effects model predicting trust ratings as a function of the deceive-inform intent manipulation (effect-coded: Inform Condition = 0.5; Deceive Condition = -0.5), the specific-approximate report manipulation (effect-coded: Approximate Condition = 0.5; Specific Condition = -0.5), and the interaction between the manipulations. Participant-level and stimuli-level random intercepts as well as participant-level and stimuli-level random slopes for the deceive-inform intent manipulation, the specific-approximate report manipulation, and the interaction between the manipulations were included as random effects. (Note that the preregistered maximal model had a singular fit, so, as preregistered, we followed the guidance of Matuschek, Kliegl,Vasishth, Baayen, & Bates (2017) and Bates, Kliegl,Vasishth, & Baayen (2018) and iteratively reduced the model until it no longer had a singular fit. The final model retained all random intercepts and slopes for participants and stimuli, but did not model any correlations between the random effects for participants.) There was a significant effect of the deceive-inform intent manipulation, *b* = 1.23, *SE* = 0.07, *t* = 16.85, *p* < 0.001, such that trust ratings were lower in the Deceive Condition than the Inform Condition, all else equal. There was a significant effect of the specific-approximate report manipulation, *b* = 0.22, *SE* = 0.05, *t* = 4.94, *p* < 0.001, such that trust ratings were higher in the Approximate Condition than the Specific Condition, all else equal. There was not a significant interaction between the two manipulations, *b* = 0.06, *SE* = 0.05, *t* = 1.30, *p* = 0.208.

#### **Correlation between truth and trust**

We preregistered that we would test the repeated-measures correlation between truth classifications and trust ratings. Truth classifications and trust ratings were positively associated (*r* = 0.580, 95% CI = [0.568, 0.592], *p* < 0.001) such that when participants classified a claim as true, they reported that they would trust other information provided by the information source to a greater extent.

### ***Non-preregistered analyses***

#### **Manipulation checks**

To ensure our manipulations affected participants’ intent ratings as expected, we tested the effect of each manipulation on its relevant manipulation-check measure.

***Intent to deceive.*** We ran a linear mixed effects models predicting participants’ intent-to-deceive ratings as a function of the deceive-inform intent manipulation (effect-coded: Inform = 0.5; Deceive = -0.5), with random slopes and intercepts. There was a significant effect of the deceive-inform intent manipulation, *b* = -1.54, *SE* = 0.09, *t* = -17.22, *p* < 0.001, such that intent-to-deceive ratings were higher in the Deceive Condition compared to the Inform Condition.

***Intent to approximate.*** We ran a linear mixed effects models predicting participants’ intent-to-approximate ratings as a function of the specific-approximate report manipulation (effect-coded: Approximate = 0.5; Specific = -0.5), with random slopes and intercepts. There was a significant effect of the specific-approximate report manipulation, *b* = 0.46, *SE* = 0.05, *t* = 9.76, *p* < 0.001, such that intent-to-approximate ratings were higher in the Approximate Condition compared to the Specific Condition.

#### **Robustness check 3: Variable true-false analysis**

While analyzing our data, we noticed that a large percentage of the participants (23.18%) classified all 24 stimuli as false. We decided to conduct a set of analyses looking only at participants who classified at least one stimulus as true and at least one stimulus as false—i.e., those who showed any within-person variance in their truth classifications. After removing participants who rated all 24 stimuli as false or all 24 stimuli as true, we were left with 391 out of the original 509 participants included in the main-text analyses.

***Truth.*** We ran a generalized linear mixed effects model predicting truth classifications as a function of the deceive-inform intent manipulation (effect-coded: Inform = 0.5; Deceive = -0.5), the specific-approximate report manipulation (effect-coded: Approximate = 0.5; Specific = -0.5), and the interaction between the manipulations. This model had the following random effects structure: participant-level and stimuli-level random intercepts as well as participant-level and stimuli-level random slopes for the deceive-inform intent manipulation, the specific-approximate report manipulation, and the interaction between the manipulations. Results from this model can be seen in Table S8a.

**Table S8a. Study 2 model results from the generalized linear mixed effects model predicting truth classifications (variable true-false analysis)**

|  | **Truth classifications** | | | | |  |
| --- | --- | --- | --- | --- | --- | --- |
| *Predictors* | *Log-Odds* | *std. Error* | *95% CI* | *z value* | *p* |  |
| (Intercept) | -2.03 | 0.20 | -2.42 – -1.63 | -10.11 | **<0.001** |  |
| inform vs deceive | 2.47 | 0.17 | 2.13 – 2.81 | 14.24 | **<0.001** |  |
| approximate vs specific | 1.16 | 0.17 | 0.82 – 1.49 | 6.81 | **<0.001** |  |
| inform vs deceive *  approximate vs specific | -0.54 | 0.28 | -1.09 – 0.02 | -1.90 | 0.057 |  |
| **Random Effects** | | | | | |  |
| σ^2^ |  | 3.29 | | | | |
| τ_00_ _subjectid_ |  | 2.33 | | | | |
| τ_00_ _stimulus_ |  | 0.70 | | | | |
| τ_11_ _subjectid.inform_vs_deceive_ |  | 2.04 | | | | |
| τ_11_ _subjectid.approximate_vs_specific_ |  | 0.56 | | | | |
| τ_11_ _subjectid.inform_vs_deceive:approximate_vs_specific_ | | 0.44 | | | | |
| τ_11_ _stimulus.inform_vs_deceive_ |  | 0.11 | | | | |
| τ_11_ _stimulus.approximate_vs_specific_ |  | 0.20 | | | | |
| τ_11_ _stimulus.inform_vs_deceive:approximate_vs_specific_ | | 0.07 | | | | |
| ρ_01_ |  | -0.46 | | | | |
|  |  | -0.59 | | | | |
|  |  | 0.57 | | | | |
|  |  | -0.19 | | | | |
|  |  | -0.28 | | | | |
|  |  | 0.73 | | | | |
| ICC |  | 0.54 | | | | |
| N _subjectid_ |  | 391 | | | | |
| N _stimulus_ |  | 24 | | | | |
| Observations |  | 9384 | | | | |
| Marginal R^2^ / Conditional R^2^ | | 0.210 / 0.633 | | | | |

***Trust.*** We ran a linear mixed effects model predicting trust classifications as a function of the deceive-inform intent manipulation (effect-coded: Inform = 0.5; Deceive = -0.5), the specific-approximate report manipulation (effect-coded: Approximate = 0.5; Specific = -0.5), and the interaction between the manipulations. This model had the following random effects structure: participant-level and stimuli-level random intercepts as well as participant-level and stimuli-level random slopes for the deceive-inform intent manipulation, the specific-approximate report manipulation, and the interaction between the manipulations. Results from this model can be seen in Table S8b.

**Table S8b. Study 2 model results from the linear mixed effects model predicting trust judgments (variable true-false analysis)**

|  | **Trust judgments** | | | | |
| --- | --- | --- | --- | --- | --- |
| *Predictors* | *Estimates* | *std. Error* | *95% CI* | *t value* | *p* |
| (Intercept) | 2.94 | 0.09 | 2.77 – 3.11 | 34.15 | **<0.001** |
| inform vs deceive | 1.28 | 0.08 | 1.13 – 1.44 | 16.16 | **<0.001** |
| approximate vs specific | 0.26 | 0.05 | 0.17 – 0.36 | 5.45 | **<0.001** |
| inform vs deceive *  approximate vs specific | 0.04 | 0.06 | -0.07 – 0.16 | 0.72 | 0.473 |
| **Random Effects** | | | | | |
| σ^2^ |  | 0.91 |  |  |  |
| τ_00_ _subjectid_ |  | 0.29 |  |  |  |
| τ_00_ _stimulus_ |  | 0.16 |  |  |  |
| τ_11_ _subjectid.inform_vs_deceive_ |  | 0.53 |  |  |  |
| τ_11_ _subjectid.approximate_vs_specific_ |  | 0.05 |  |  |  |
| τ_11_ _subjectid.inform_vs_deceive:approximate_vs_specific_ | | 0.11 |  |  |  |
| τ_11_ _stimulus.inform_vs_deceive_ |  | 0.11 |  |  |  |
| τ_11_ _stimulus.approximate_vs_specific_ |  | 0.04 |  |  |  |
| τ_11_ _stimulus.inform_vs_deceive:approximate_vs_specific_ | | 0.04 |  |  |  |
| ρ_01_ |  | 0.04 |  |  |  |
|  |  | -0.14 |  |  |  |
|  |  | -0.36 |  |  |  |
|  |  | -0.24 |  |  |  |
|  |  | -0.02 |  |  |  |
|  |  | -0.20 |  |  |  |
| N _subjectid_ |  | 391 |  |  |  |
| N _stimulus_ |  | 24 |  |  |  |
| Observations |  | 9384 |  |  |  |
| Marginal R^2^ / Conditional R^2^ |  | 0.320 / NA | |  |  |

#### **Robustness check 4: Persons-as-effect-sizes analysis**

As recommended by McManus and colleagues (2023) and Grice and colleagues (2020), we tested how many participants showed our expected pattern of results for the effect of the deceive-inform intent manipulation, the specific-approximate report manipulation, and the interaction between the two on truth classifications. As reported in the main text, there were significant mean-level differences in truth classifications between the Inform Condition and the Deceive Condition and between the Approximate Condition and the Specific Condition. Additionally, there was a borderline-significant interaction between the deceive-inform intent manipulation, the specific-approximate report manipulation. However, it is possible that a minority of participants could be responsible for these effects if, for example, some participants showed the effect very strongly, but most participants showed no effect (McManus et al., 2023).

In order to calculate the number of participants who showed the predicted effect of the deceive-inform intent manipulation, we considered participants as (a) showing the predicted effect if they classified more stimuli as true in the Inform Condition than they did in the Deceive Condition, (b) showing no effect if they classified the same number of stimuli as true in the Inform Condition as they did in the Deceive Condition, or (c) showing the unpredicted effect if they classified more stimuli as true in the Deceive Condition than they did in the Inform Condition. 333 participants (65.4%) showed the predicted effect, 139 participants (27.3%) showed no effect, and 37 (7.3%) participants showed the unpredicted effect. Thus, the predicted pattern was the modal pattern and almost two-thirds of participants (65.4%) showed this pattern.

In order to calculate the number of participants who showed the predicted effect of the specific-approximate report manipulation, we considered participants as (a) showing the predicted effect if they classified more stimuli as true in the Approximate Condition than they did in the Specific Condition, (b) showing no effect if they classified the same number of stimuli as true in the Approximate Condition as they did in the Specific Condition, or (c) showing the unpredicted effect if they classified more stimuli as true in the Specific Condition than they did in the Approximate Condition. 251 participants (49.3%) showed the predicted effect, 178 participants (35.0%) showed no effect, and 80 participants (15.7%) showed the unpredicted effect. Thus, the predicted pattern was the modal pattern and roughly half of participants (49.3%) showed this pattern.

In order to calculate the number of participants who showed the observed interaction effect between the deceive-inform intent manipulation and the specific-approximate report manipulation, we calculated the number of participants who showed the full directional pattern of results suggested by the observed interaction effect, as recommended by McManus and colleagues (2023). This pattern consists of (1) the simple effect of participants classifying more claims as true in the Inform Condition versus the Deceive Condition when the information source provided a rounded number (Approximate Condition); (2) the simple effect of participants classifying more claims as true in the Inform Condition versus the Deceive Condition when the information source provided a non-rounded number (Specific Condition); (3) a positive difference between the simple effects such that the first simple effect is larger than the second simple effect. This analysis revealed that 98 out of 509 participants (19.3%) showed all three directional patterns. This represented the second-most common pattern of the two simple effects and the difference between the simple effects. While 19.3% does not seem negligible, it is not a majority of participants and it did not represent the modal pattern. We remain agnostic about the importance of this degree of prevalence, but hope that this calculation can help contextualize the observed borderline interaction. If nothing else, we believe this result presents an interesting opportunity for future work to explore individual differences in people’s sensitivity to intent when classifying claims as true or false. After all, this interaction suggests that Gricean maxims (i.e., an assumed intent to provide an approximate or precise report) might, for some people, moderate whether intentions to inform or deceive affect how people classify the veracity of claims.

Table S9 shows the joint pattern of results for the person-level effect sizes of both manipulations. Figure S4 shows the percentage of participants whose responses show each of the 13 possible directional simple effect patterns.

**Table S9. Number of participants in Study 2 who showed the predicted pattern of results for each manipulation**

|  |  | **Specific-Approximate Report Manipulation** | | |  |
| --- | --- | --- | --- | --- | --- |
|  |  | Predicted  effect | No  effect | Unpredicted effect | *Total* |
| **Deceive-Inform Intent Manipulation** | Predicted effect | 214  (42.0%) | 50  (9.8%) | 69  (13.6%) | *333*  *(65.4%)* |
|  | No  effect | 12  (2.4%) | 125  (24.6%) | 2  (0.3%) | *139*  *(27.3%)* |
|  | Unpredicted effect | 25  (4.9%) | 3  (0.6%) | 9  (1.8%) | *37*  *(7.3%)* |
|  | *Total* | *251*  *(49.3%)* | *178*  *(35.0%)* | *80*  *(15.7%)* |  |

The number (and associated percentage) of participants who showed the various possible patterns of truth classifications among the 24 stimuli they evaluated. For the deceive-inform intent manipulation, participants were considered as (a) showing the predicted effect if they classified more stimuli as true in the Inform Condition than they did in the Deceive Condition, (b) showing no effect if they classified the same number of stimuli as true in the Inform Condition as they did in the Deceive Condition, or (c) showing the unpredicted effect if they classified more stimuli as true in the Deceive Condition than they did in the Inform Condition. For the specific-approximate report manipulation, participants were considered as (a) showing the predicted effect if they classified more stimuli as true in the Approximate Condition than they did in the Specific Condition, (b) showing no effect if they classified the same number of stimuli as true in the Approximate Condition as they did in the Specific Condition, or (c) showing the unpredicted effect if they classified more stimuli as true in the Specific Condition than they did in the Approximate Condition. Text in italics shows the row or column total.


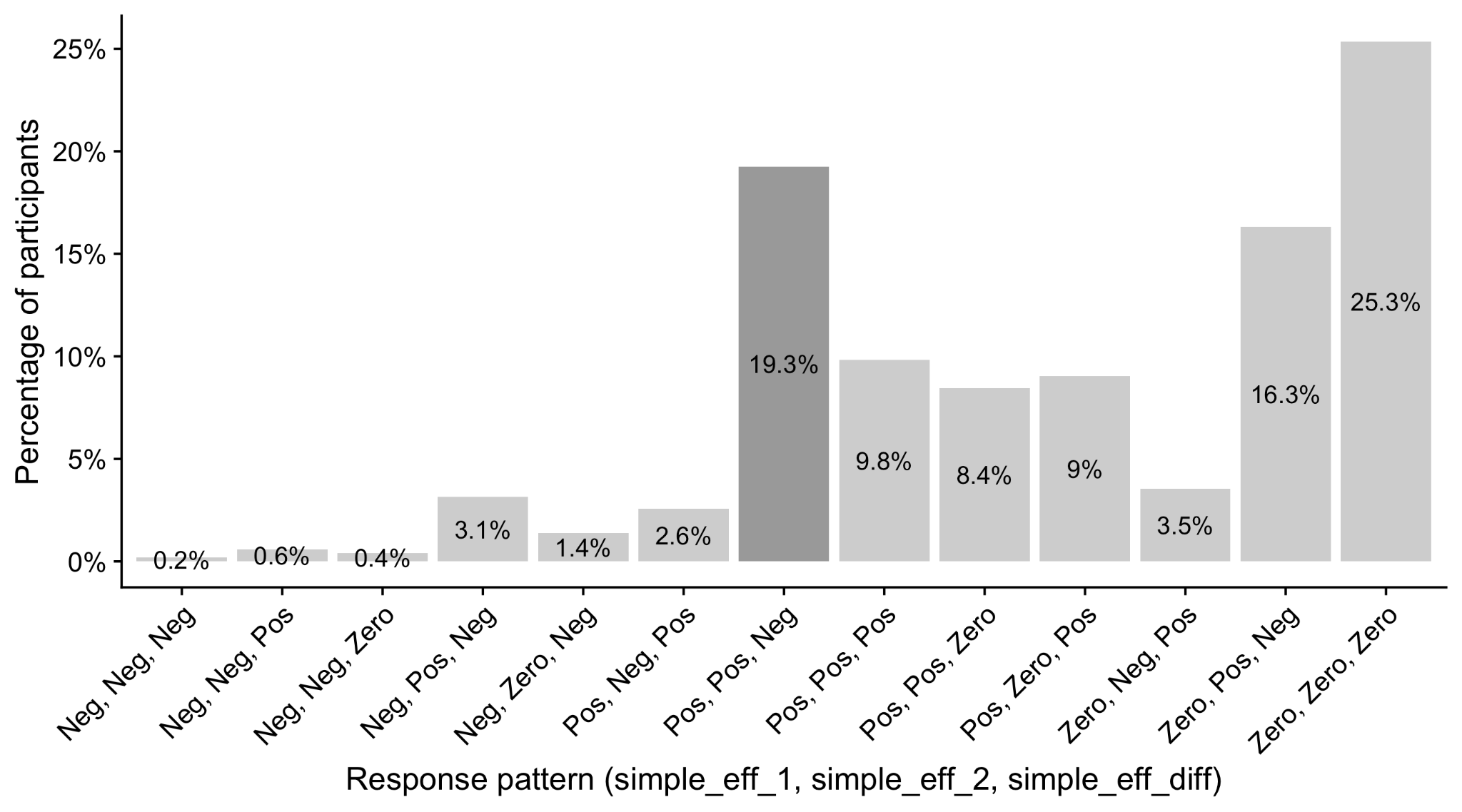


**Fig. S4.** Study 2 person-level response patterns for the 2x2 interaction between the deceive-inform intent manipulation and the specific-approximate report manipulation. The x-axis values refer to each of the 13 possible directional patterns representing the following: **(1)** **Simple Effect 1**: the number of claims classified as true in the Inform Condition minus the number classified as true in the Deceive Condition when the information source provided a rounded number (Approximate Condition); **(2) Simple Effect 2:** the number of claims classified as true in the Inform Condition minus the number classified as true in the Deceive Condition when the information source provided a non-rounded number (Specific Condition); **(3) Difference between Simple Effect 1 and Simple Effect 2:** Simple Effect 1 minus Simple Effect 2. “Pos” refers to a positive value; “Neg” refers to a negative value; “Zero” refers to a value of zero. The pattern “Pos, Pos, Neg” has a darker bar to indicate that this was the pattern implied by the observed borderline-significant interaction effect in Study 2.

#### **Correlations between truth and intent ratings**

We conducted exploratory repeated-measures correlations assessing the relationships between truth classifications and both types of intent ratings, across the full sample and within each condition. The repeated-measures correlations between truth classifications and intent-to-deceive ratings—across conditions and within condition—were all statistically significant at *p* < 0.001 and ranged from *r* = -0.406 to *r* = -0.468 such that the more intent-to-deceive participants attributed to the information source, the less likely they were to classify the claim as true. Similarly, the repeated-measures correlations between truth classifications and intent-to-approximate ratings—across conditions and within condition—were all statistically significant at *p* < 0.001 and ranged from *r* = 0.441 to *r* = 0.522 such that the more intent-to-approximate participants attributed to the information source, the more likely they were to classify the claim as true. The results for each correlation are displayed in the following table.

**Table S10. Study 2 repeated-measures correlations**

|  | Correlation between truth classifications and intent-to-deceive ratings | Correlation between truth classifications and intent-to-approximate ratings |
| --- | --- | --- |
| Across all conditions | *r* = -0.468 | *r* = 0.493 |
| Within the Inform Condition | *r* = -0.416 | *-* |
| Within the Deceive Condition | *r* = -0.406 | *-* |
| Within the Approximate Condition | *-* | *r* = 0.522 |
| Within the Specific Condition | *-* | *r* = 0.441 |

Repeated-measures correlations between truth classifications and intent-to-deceive ratings and between truth classifications and intent-to-approximate ratings. Correlations were tested both across all conditions, as well as within each condition.

#### **Order of truth and trust measures**

In an exploratory analysis, we assessed whether the order in which participants answered the truth and trust measures (the order of which was randomized for every participant on each stimulus) affected truth classifications. To test this, we ran a generalized linear mixed effects model predicting truth classifications as a function of measure order (truth-first = 1; trust-first = 0), with random intercepts and slopes for both participants and stimuli.

We did not find a significant effect of the order in which participants responded to the truth and trust measures, *b* = -0.07 (OR = 0.93, OR 95% CI [0.80 – 1.08]), *SE* = 0.08, *z* = -0.93, *p* = 0.352.

#### **Effect of discrepancy between claim and ground truth**

In an exploratory analysis, we assessed whether the degree of numerical discrepancy between the claim of fact and the supplied ground truth in each stimulus affected truth classifications and/or moderated the effects of any of the manipulations.

To test this, we calculated the discrepancy between the ground truth number and the number each participant saw in the claim of fact as a percentage of the ground truth number. So, for example, if the ground truth number was 463 and the participant learned that the information source claimed the number was 499, we took the difference between the numbers (36 in this case) and calculated what percentage of the ground truth number this represented (7.8% in this case). This served as the measure of discrepancy.

We then ran a generalized linear mixed effects model predicting truth classifications (1 = True; 0 = False) as a function of: the deceive-inform intent manipulation (effect-coded: Inform Condition = 0.5; Deceive Condition = -0.5); the specific-approximate report manipulation (effect-coded: Approximate Condition = 0.5; Specific Condition = -0.5); discrepancy; the two-way interaction between the deceive-inform intent manipulation and specific-approximate report manipulation; the two-way interaction between the deceive-inform intent manipulation and discrepancy; the two-way interaction between the specific-approximate report manipulation and discrepancy; and the three-way interaction between the deceive-inform intent manipulation, the specific-approximate report manipulation, and discrepancy. Participant-level and stimuli-level random intercepts were included as random effects. Results from this model can be seen in Table S11.

There was a statistically significant effect of both of the condition manipulations, in line with the results from the primary analysis reported in the main manuscript. Unlike the primary analysis, however, there was no statistically significant effect of the interaction between the two manipulations. Additionally, there was a statistically significant effect of discrepancy, such that participants were less likely to classify the claim as true as the discrepancy increased. Moreover, there was a statistically significant effect of the interaction between the specific-approximate report manipulation and discrepancy, such that the effect of the specific-approximate report manipulation on truth classifications diminished as the discrepancy between the ground truth and the report increased. In other words, the interaction effect suggests that whether an information source reported a rounded number or non-rounded number affected participants’ truth classifications less when there was a large discrepancy between the ground truth and the report. We did not find evidence of any other moderating effects of discrepancy.

**Table S11. Study 2 model results from the generalized linear mixed effects model predicting truth classifications (discrepancy analysis)**

|  | **Truth Classifications** | | | | |
| --- | --- | --- | --- | --- | --- |
| *Predictors* | *Log-Odds* | *std. Error* | *95% CI* | *z value* | *p* |
| (Intercept) | -1.87 | 0.33 | -2.51 – -1.24 | -5.76 | **<0.001** |
| inform vs deceive | 1.79 | 0.14 | 1.52 – 2.07 | 12.67 | **<0.001** |
| approximate vs specific | 1.16 | 0.14 | 0.89 – 1.44 | 8.27 | **<0.001** |
| discrepancy | -0.06 | 0.03 | -0.11 – -0.01 | -2.34 | **0.019** |
| inform vs deceive * approximate vs specific | 0.45 | 0.28 | -0.09 – 1.00 | 1.63 | 0.104 |
| inform vs deceive * discrepancy | 0.01 | 0.01 | -0.01 – 0.04 | 0.88 | 0.379 |
| approximate vs specific * discrepancy | -0.03 | 0.01 | -0.06 – -0.00 | -2.06 | **0.040** |
| inform vs deceive * approximate vs specific * discrepancy | -0.03 | 0.02 | -0.08 – 0.02 | -1.20 | 0.230 |
| **Random Effects** | | | | | |
| σ^2^ | 3.29 | | | | |
| τ_00_ _subjectid_ | 3.62 | | | | |
| τ_00_ _stimulus_ | 0.58 | | | | |
| ICC | 0.56 | | | | |
| N _subjectid_ | 509 | | | | |
| N _stimulus_ | 24 | | | | |
| Observations | 12216 | | | | |
| Marginal R^2^ / Conditional R^2^ | 0.133 / 0.619 | | | | |

# **Supplementary Studies**

## **Study S1**

### ***Methods***

#### **Participants**

150 U.S. participants—75 Democrats and 75 Republicans—were recruited on Amazon Mechanical Turk via CloudResearch Panels. Per our preregistered exclusion criteria, 18 participants were excluded for not completing the primary dependent measures, taking the study more than once, failing the attention check, and/or indicating discrepant political affiliations. Our final sample size was 132 (50.0% Democrat, 50.0% Republican; 51.5% female, 48.5% male; *M_age_* = 43.93).

#### **Materials and procedures**

Participants saw four stimuli. Each stimulus contained a real scientific finding (e.g., “57 individuals, out of just over 2 million patients, developed temporary heart complications after having received at least one dose of a COVID-19 vaccine”) and an ostensibly real (although actually fabricated) report from a news outlet about that scientific finding (e.g., “a study on the COVID-19 vaccine found that dozens of individuals developed heart complications after receiving at least one dose of the COVID-19 vaccine.”). Supplying participants with the scientific finding on which the report was based ensured that all participants knew the ground truth of the topic the report detailed. Each report was crafted to have a slant that supported the political agenda of either U.S. Republicans (as in the previous example) or U.S. Democrats. There were two Republican-slant stimuli and two Democrat-slant stimuli in total (See Table S12 for the complete list of stimuli).

For each news report, participants were either told that it came from a “conservative-leaning” or “liberal-leaning” news outlet. The ostensible political leaning of the news outlet was counterbalanced such that each participant saw one conservative-slant stimulus attributed to a conservative-leaning outlet and one attributed to a liberal-leaning outlet. The same held for the two Democrat-slant stimuli. The order of the stimuli was also randomized and counterbalanced such that, across participants, each stimulus was assigned in each of the four order slots, and each stimulus was attributed to both conservative-leaning outlets and liberal-leaning outlets.

The alignment between the political leaning and the outlet constituted our two conditions. When a stimulus was in the Aligned Condition, the political leaning of the news outlet matched the political slant of the report (conservative-Republican or liberal-Democrat pairings). When a stimulus was in the Misaligned Condition, the political leaning of the news outlet did not match the political slant of the report (conservative-Democrat or liberal-Republican pairings).

After reading each stimulus, participants completed several measures. Our primary dependent variables were as follows. The truth classification measure asked, “Assuming the original scientific finding is correct, would you consider the report from this news outlet to be true or false?” (1 = True, 0 = False). The accuracy measure asked, “Assuming the original scientific finding is correct, how accurate would you consider the report from the news outlet to be?” (1 = Not at all, 5 = A great deal). The accuracy intentions measure asked, “To what degree do you think the news outlet was trying to accurately report the scientific finding?” (1 = Not at all, 5 = A great deal).

We also included a few measures to assess features of the stimuli that might allow us to improve them for future studies. Two measures assessing perceived political leaning of the scientific finding and of the news report asked, “How much do you think the original scientific finding supports a conservative political agenda versus a liberal political agenda in the U.S.?” and “How much do you think the report made by the news outlet supports a conservative political agenda versus a liberal political agenda in the U.S.?” (1 = Strongly supports conservative agenda, 7 = Strongly supports liberal agenda). A measure of belief in the scientific finding asked, “Do you believe the original scientific finding?” (1 = Yes, 0 = Unsure, -1 = False). Participants were also asked to explain their truth classification in an open-response box.

At the end of the study, participants were informed that the news reports were made up, but that the scientific findings they read were real.

We expected that when the political leaning of the news outlet aligned with the political slant of the report (Aligned Condition), participants would attribute lower intent-to-be-accurate ratings to the news outlet than when the political leaning of the news outlet did not align with the political slant of the report (Misaligned Condition). In turn, we hypothesized that participants would be less likely to classify the report as true in the Aligned Condition compared to the Misaligned Condition.

**Table S12. Study S1 Stimuli**

| **Stimulus number** | **Stimulus** | **Political Slant** |
| --- | --- | --- |
| 1 | **Original scientific finding**: There is about a 2 in 10 chance that human activity has affected tropical storm activity.    **News report about scientific finding**: A [conservative-leaning / liberal-leaning] news outlet stated, “it is not at all likely that human activity has affected tropical storm activity.” | Republican |
| 2 | **Original scientific finding**: 57 individuals, out of just over 2 million patients, developed temporary heart complications after having received at least one dose of a COVID-19 vaccine.    **News report about scientific finding**: A [conservative-leaning / liberal-leaning] news outlet stated, “a study on the COVID-19 vaccine found that dozens of individuals developed heart complications after receiving at least one dose of the COVID-19 vaccine.” | Republican |
| 3 | **Original scientific finding**: Between August 31st and September 6th, 369 children in the U.S.—about 0.0005% of the youth population—were hospitalized per day, on average, with COVID-19.    **News report about scientific finding**: A [conservative-leaning / liberal-leaning] news outlet stated, “Between August 31st and September 6th, hundreds of children were being hospitalized every day, on average, with COVID-19.” | Democrat |
| 4 | **Original scientific finding**: People who experience daily discrimination have a 22% increased risk of death and people who do little or no vigorous exercise have a 15% increased risk of death.    **News report about scientific finding**: A [conservative-leaning / liberal-leaning] news outlet stated, “experiencing daily discrimination puts people at much higher risk of dying compared to not exercising.” | Democrat |

Stimuli used in Study S1. The bracketed text indicates the information that was manipulated depending on the condition in which participants saw each stimulus. For each stimulus, participants saw the bolded labels of “Original scientific finding” and “News report about scientific finding,” as depicted in the second column. The political slant of the news report is indicated in the third column.

### ***Results***

#### **Primary analyses (preregistered)**

We ran a generalized linear mixed effects model predicting the truth measure and linear mixed effects models predicting the accuracy and accuracy intentions measures. Each model included the same fixed-effects predictors: condition (effect-coded: aligned = 0.5; misaligned = -0.5), participant political party (effect-coded: Democrat = 0.5; Republican = -0.5), and the interaction between condition and participant political party. Each model contained random intercepts for participants.

***Intent-to-be-accurate judgments (full sample).*** We did not observe a significant effect of condition, *b =* -0.20*, SE =* 0.11*, t =* -1.88*, p* = 0.061, participant political party, *b =* -0.05*, SE =* 0.14*, t =* -0.36*, p* = 0.722, or the interaction term, *b = 0.22, SE =* 0.21*, t =* 1.03*, p* = 0.305.

***Truth classifications (full sample).*** We did not observe a significant effect of condition, *b =* -0.10*, SE =* 0.18*, z =* -0.54*, p* = 0.590, participant political party, *b =* 0.10*, SE =* 0.19*, z =* 0.51*, p* = 0.612, or the interaction term, *b =* -0.001*, SE =* 0.36*, z =* -0.00*, p* = 0.997.

***Accuracy Judgments (full sample).*** We did not observe a significant effect of condition, *b =* -0.21*, SE =* 0.11*, t =* -1.91*, p* = 0.058, participant political party, *b =* 0.16*, SE =* 0.14*, t =* 1.18*, p* = 0.240, or the interaction term, *b =* 0.14*, SE =* 0.22*, t =* 0.66*, p* = 0.511.

#### **Secondary analyses (preregistered)**

***Truth classifications (2x2x2 analysis; full sample).*** Another way of modeling the effects of our manipulations on truth classifications, while controlling for participants’ political affiliations is treat this study as a 2x2x2 in which the political slant of the news report (Republican = -0.5, Democrat = 0.5), the political-leaning of the news outlet (conservative = -0.5, liberal = 0.5), and the political affiliation of the participant (Republican = -0.5, Democrat = 0.5) are each treated as a separate factor. Although our primary hypotheses were concerned with the political alignment between the news outlet and the report, we preregistered that we would also model the data using this 2x2x2 framework in case it uncovered some unpredicted interactions between political party and either the slant of the news report or the leaning of the outlet. The full output from this analysis can be seen in Table S13.

**Table S13. Study S1 model output from the generalized linear mixed effects model predicting truth classifications using the 2x2x2 approach**

|  | **Truth classifications** | | | | |
| --- | --- | --- | --- | --- | --- |
| *Predictors* | *Log-Odds* | *std. Error* | *95% CI* | *z value* | *p* |
| (Intercept) | 0.36 | 0.10 | 0.17 – 0.56 | 3.63 | **<0.001** |
| report slant | 0.53 | 0.18 | 0.17 – 0.89 | 2.89 | **0.004** |
| outlet leaning | 0.17 | 0.18 | -0.19 – 0.53 | 0.92 | 0.356 |
| participant politics | 0.12 | 0.20 | -0.27 – 0.50 | 0.59 | 0.555 |
| report slant *  outlet leaning | -0.17 | 0.37 | -0.89 – 0.55 | -0.47 | 0.641 |
| report slant *  participant politics | 0.74 | 0.37 | 0.02 – 1.46 | 2.02 | **0.043** |
| outlet leaning *  participant politics | 0.41 | 0.37 | -0.31 – 1.12 | 1.10 | 0.269 |
| report slant * outlet leaning * participant politics | 0.05 | 0.73 | -1.39 – 1.48 | 0.07 | 0.948 |
| **Random Effects** | | | | | |
| σ^2^ | 3.29 | | | | |
| τ_00_ _subjectid_ | 0.18 | | | | |
| ICC | 0.05 | | | | |
| N _subjectid_ | 132 | | | | |
| Observations | 528 | | | | |
| Marginal R^2^ / Conditional R^2^ | 0.036 / 0.085 | | | | |

Model output from the generalized linear mixed effects model predicting truth classifications as a function of the political slant of the news report (Republican, Democrat), the political-leaning of the news outlet (conservative, liberal), the political affiliation of the participant (Republican, Democrat), and all 2-way and 3-way interactions, with random intercepts for participants.

***Correlation between truth classifications and accuracy judgments by condition (full sample).*** As preregistered, we tested the correlation between truth classifications and accuracy judgments, by condition. After preregistering this analysis, we realized that a repeated-measures correlation is more appropriate given that participants provided multiple ratings. We report both the correlation and repeated-measures correlation results in the table below.

**Table S14. Study S1 correlations between truth and accuracy, by condition**

| **Condition** | **Pearson’s product moment correlation between**  **truth and accuracy** | **Repeated-measures correlation between truth and accuracy** |
| --- | --- | --- |
| aligned | *r* = 0.675 | *r* = 0.699 |
| misaligned | *r* = 0.704 | *r* = 0.690 |

#### **Exploratory analyses (not preregistered)**

Given that the effect of condition on accuracy intention judgments was not significant, we could not conclude that manipulating the alignment between the news outlet’s political leaning and the political skew of its report affected intent attributions. Thus, in order to better assess the relationship between intent judgments and truth classifications, we conducted a few non-preregistered follow-up analyses. First, we tested the repeated-measures correlation between intent-to-be-accurate judgments and truth classifications in four different configurations of the data: the full sample (n=132), only those for whom the manipulation was successful (n=60), only those for whom the manipulation was unsuccessful (n=72), and only for the observations for which participants reported that they believed the scientific finding (257 observations). Second, we re-ran our preregistered primary analyses on only the participants for whom the manipulation was successful (n=60). We defined the manipulation as being successful for a participant if their mean intent-to-be-accurate judgment score was lower in the aligned condition than in the misaligned condition. We defined the manipulation as unsuccessful for a participant if their mean accuracy intention judgment score was greater than or equal in the aligned condition compared to the misaligned condition.

***Correlation between intent-to-be-accurate judgments and truth classifications.*** We observed a repeated-measures correlation between intent-to-be-accurate judgments and truth classifications of *r* = 0.650, 95% CI = [0.589, 0.703], *p* < .001 in the full sample, *r* = 0.656, 95% CI = [0.563, 0.732], *p* < .001 in the manipulation-success sample, *r* = 0.647, 95% CI = [0.562, 0.718], *p* < .001 in the manipulation-failure sample, and *r* = 0.685, 95% CI = [0.587, 0.763], *p* < .001 among the observations for which participants believed the scientific finding. All four correlations were similar in magnitude and positive such that participants who rated an outlet as having greater intentions to be accurate were more likely to classify the outlet’s report as true.

***Intent-to-be-accurate judgments (manipulation-success sample).*** Among the sample of participants for whom the manipulation was successful (n=60), we observed a significant effect of condition, *b =* -1.32*, SE =* 0.14*, t =* -9.34*, p* < 0.001, such that predicted accuracy intentions ratings were higher in the misaligned condition versus the aligned condition, all else equal. We did not observe a significant effect of participant political party, *b =* -0.33*, SE =* 0.20*, t =* -1.69*, p* = 0.097, or of the interaction term, *b =* 0.13*, SE =* 0.28*, t =* 0.47*, p* = 0.639.

***Truth classifications (manipulation-success sample).*** Among the sample of participants for whom the manipulation was successful (n=60)*,* we observed a significant effect of condition, *b =* -1.31*, SE =* 0.30*, z =* -4.35*, p* < 0.001, such that participants in the misaligned condition were more likely to classify the report as true, all else equal. We did not observe a significant effect of participant political party, *b =* 0.19*, SE =* 0.33*, z =* 0.59*, p* = 0.556, or the interaction term, *b =* -0.25*, SE =* 0.58*, z =* -0.43*, p* = 0.670.

***Accuracy Judgments (manipulation-success sample).*** Among the sample of participants for whom the manipulation was successful (n=60)*,* we observed a significant effect of condition, *b =* -1.16*, SE =* 0.14*, t =* -8.30*, p* < 0.001, such that predicted accuracy intentions ratings were higher in the misaligned condition versus the aligned condition, all else equal. We did not observe a significant effect of either participant political affiliation, *b =* -0.07*, SE =* 0.20*, t =* -0.35*, p* = 0.725, or the interaction term, *b =* 0.29*, SE =* 0.28*, t =* 1.03*, p* = 0.303.


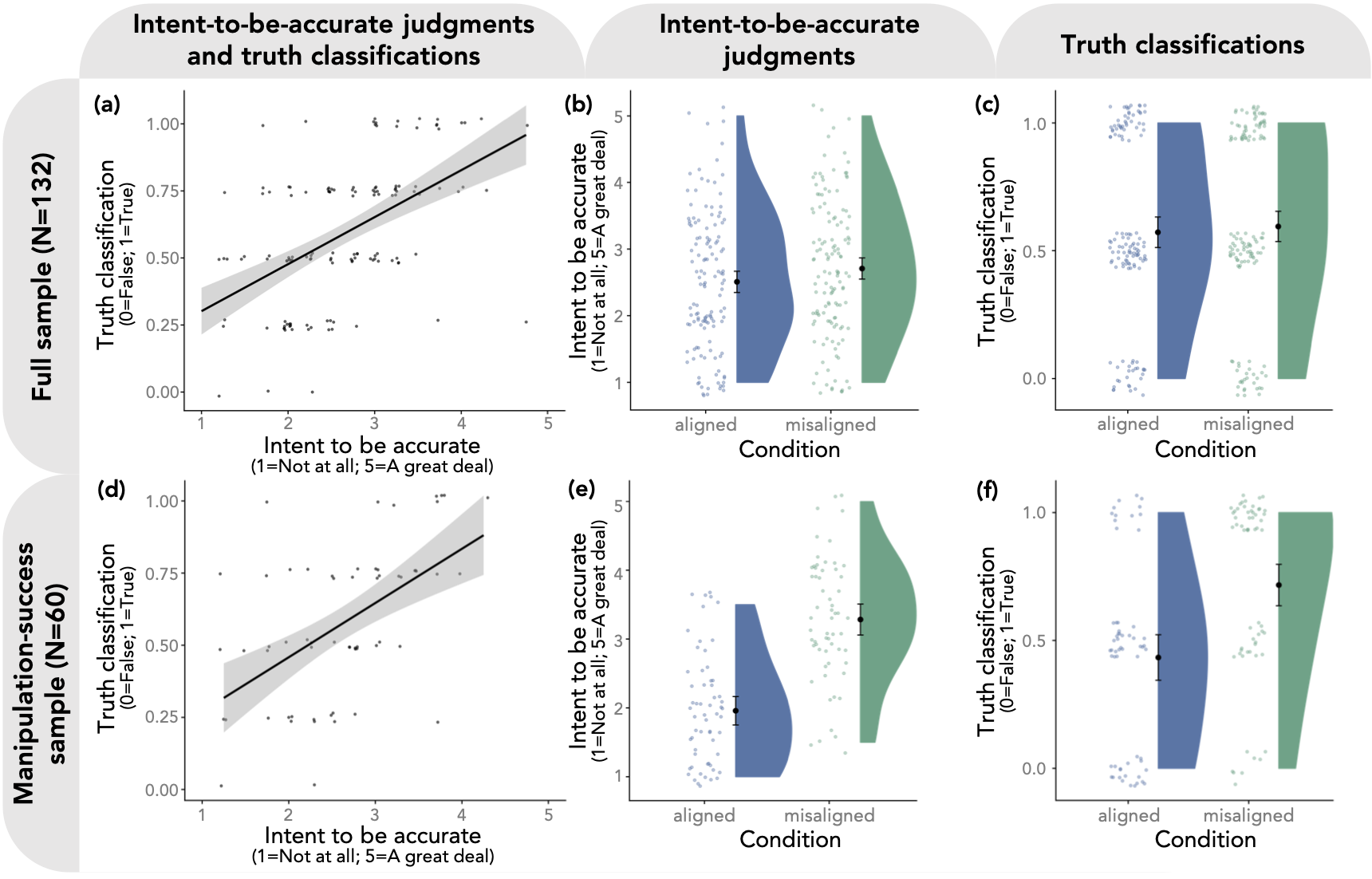


**Fig. S5.** Study S1 results. (a) Scatterplot between intent-to-be-accurate judgments and truth classifications with best-fit linear line, among the full sample (N=132); (b) Intent-to-be-accurate judgments, by condition, among the full sample (N=132); (c) Truth classifications, by condition, among the full sample (N=132); (d) Scatterplot between intent-to-be-accurate judgments and truth classifications with best-fit linear line, among the manipulation-success sample (N=60); (e) Intent-to-be-accurate judgments, by condition, among the manipulation-success sample (N=60); (f) Truth classifications, by condition, among the manipulation-success sample (N=60). In (a)-(f), each individual data-point represents all observations for each participant in the specified condition averaged together (i.e., a person-level condition mean), larger dots with error bars represent condition-level means, and error bars represent 95% confidence intervals.

## **Study S2A**

### ***Methods***

#### **Participants**

180 U.S. participants were recruited on Amazon Mechanical Turk. Per our preregistered exclusion criteria, 9 participants were excluded for failing the pre-manipulation attention check or not completing the primary dependent measure. Note that we also preregistered that we would also exclude any participants who took the study more than once as determined by their Amazon Mechanical Turk worker ID. We mistakenly did not collect participants’ worker IDs, so we were unable to apply this exclusion criterion. Our final sample size was 171 (47.4% female, 51.5% male, 0.6% non-binary/other, 0.6% undisclosed; *M_age_* = 40.89).

#### **Materials and procedures**

Participants were assigned to one of two conditions. Each participant read the following stimulus, where the bracketed section indicates the information that was manipulated between conditions:

*23,110 people attended a politician’s rally. A journalist reported that [23,000 / 23,104] people attended the rally.*

Participants in the Approximate Condition learned that the journalist reported a rounded number (23,000), while participants in the Specific Condition learned that the journalist reported a non-rounded number (23,104). The number in the Specific Condition (23,104) was designed to be numerically closer to the ground truth (23,110) than the number in the Approximate Condition (23,000).

After reading the stimulus, the only measure participants responded to was a truth classification measure asking, “Would you consider the information reported by the journalist to be true or false?” (True = 1, False = 0).

We predicted that participants would be more likely to classify the information reported by the journalist as true in the Approximate Condition versus the Specific Condition.

### ***Results***

#### **Preregistered analyses**

To test whether participants would be more likely to classify the information reported by the journalist as true in the Approximate Condition versus the Specific Condition, we ran a logistic regression, predicting truth classification (false = 0; true = 1) as a function of condition (specific = 0; approximate = 1).

***Truth classification.*** We did not observe a significant effect of condition, *b* = 0.67, *SE* = 0.38, *z* = 1.78, *p* = 0.075.


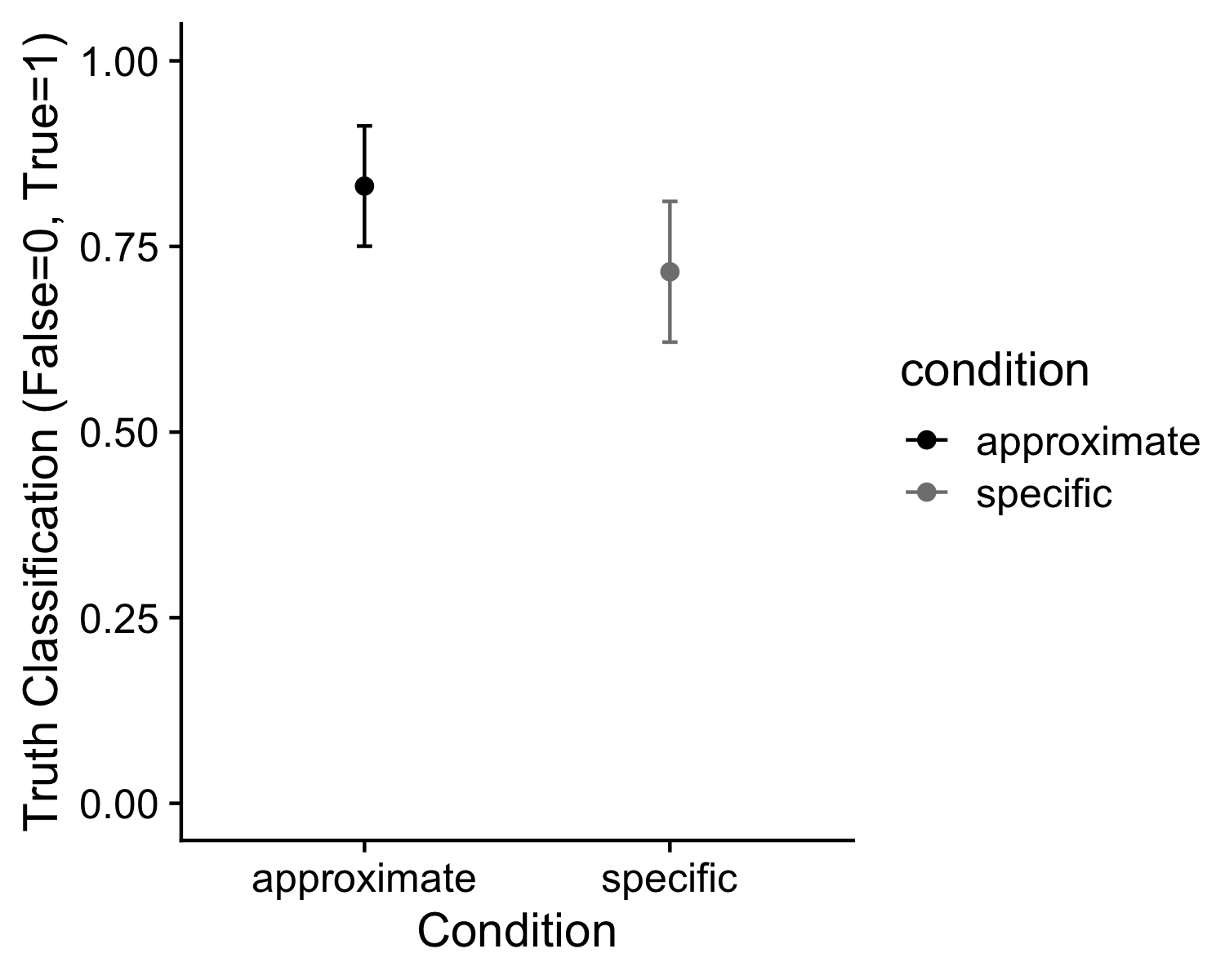


**Fig. S6.** Mean truth classifications, by condition (N=171). Error bars represent 95% confidence intervals.

## **Study S2B**

### ***Methods***

#### **Participants**

420 U.S. participants were recruited on Amazon Mechanical Turk. Per our preregistered exclusion criteria, 27 participants were excluded for failing the pre-manipulation attention check, not completing the primary dependent measure, and/or taking the study more than once. Our final sample size was 393 (45.0% female, 53.9% male, 0.3% non-binary/other, 0.8% undisclosed; *M_age_* = 42.44).

#### **Materials and procedures**

Participants were assigned to one of two conditions. Each participant read the following stimulus, where the bracketed section indicates the information that was manipulated between conditions:

*23,890 people attended a politician’s rally. A journalist reported that [24,000 / 23,896] people attended the rally.*

Participants in the Approximate Condition learned that the journalist reported a rounded number (24,000), while participants in the Specific Condition learned that the journalist reported a non-rounded number (23,896). The number in the Specific Condition (23,896) was designed to be numerically closer to the ground truth (23,890) than the number in the Approximate Condition (24,000).

The only substantive difference between this stimulus and the one employed in Study S2A is that the number reported by the journalist is higher than the true number in both conditions (rather than lower than the true number in both conditions). In Study S2A, a high percentage of participants in the Specific Condition classified the information from the journalist as true. We were concerned that this could be due to some participants thinking that the journalist’s report of 23,104 rally attendees (when the real number was 23,110) was *technically* true since there were indeed 23,104 rally attendees, there were also just 6 more attendees as well.

After reading the stimulus, participants responded to a truth classification measure asking, “Would you consider the information reported by the journalist to be true or false?” (True = 1, False = 0). Then, on the following page of the survey, participants reported why they considered this information true or false in an open-response question.

As in Study S2A, we predicted that participants would be more likely to classify the information reported by the journalist as true in the Approximate Condition versus the Specific Condition.

### ***Results***

#### **Preregistered analyses**

To test whether participants would be more likely to classify the information reported by the journalist as true in the Approximate Condition versus the Specific Condition, we ran a generalized linear regression, predicting truth classification as a function of condition.

***Truth classification.*** We did not observe a significant effect of condition, *b* = 0.31, *SE* = 0.23, *z* = 1.35, *p* = 0.177.

**
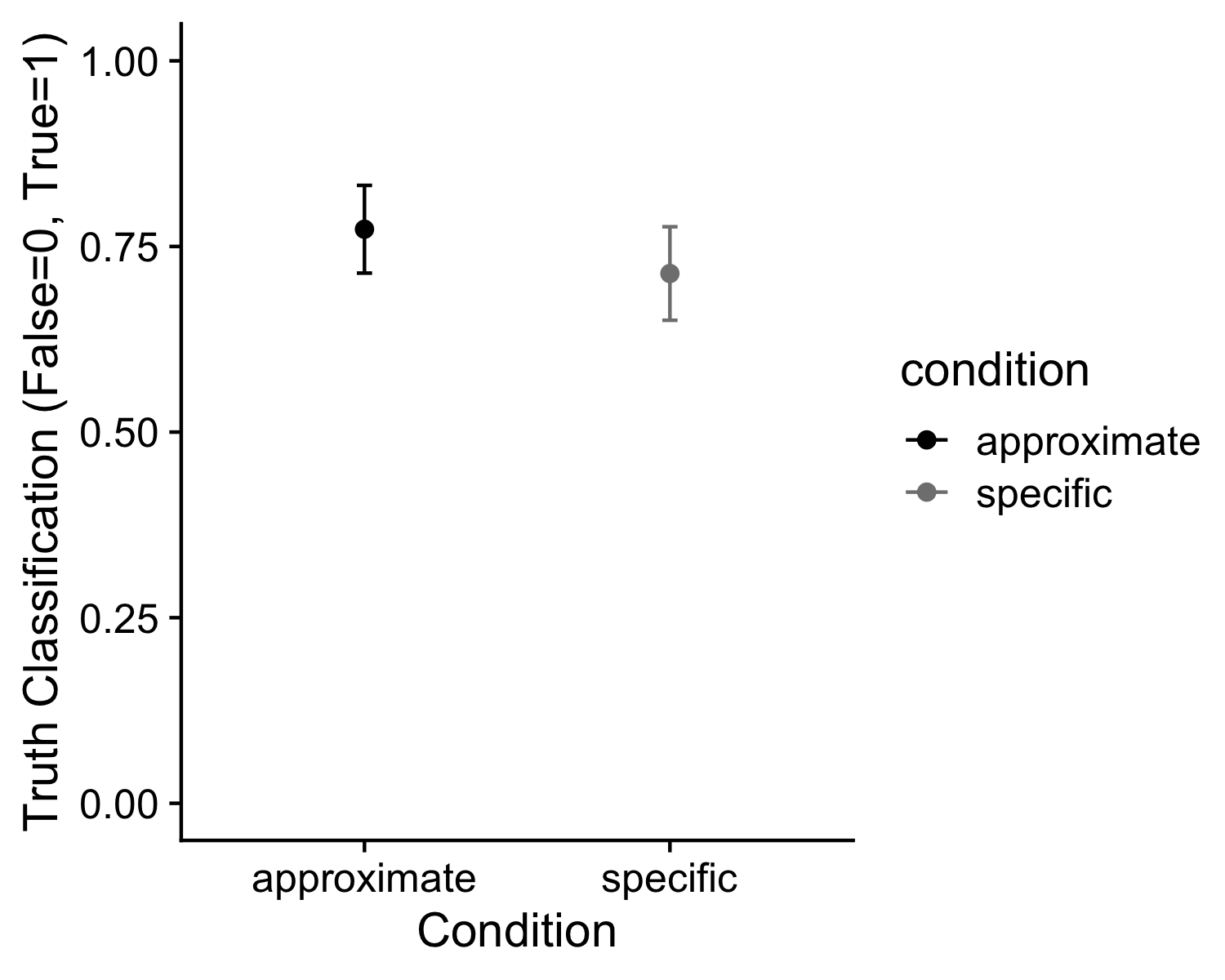
**

**Fig. S7.** Mean truth classifications, by condition (N=393). Error bars represent 95% confidence intervals.

## **Study S2C**

### ***Methods***

#### **Participants**

410 U.S. participants were recruited on Amazon Mechanical Turk. Per our preregistered exclusion criteria, 24 participants were excluded for failing the pre-manipulation attention check, not completing the primary dependent measure, and/or taking the study more than once. Our final sample size was 386 (49.2% female, 49.2% male, 0.8% non-binary/other, 0.8% undisclosed; *M_age_* = 41.56).

#### **Materials and procedures**

Participants were assigned to one of two conditions. Each participant read the following stimulus, where the bracketed section indicates the information that was manipulated between conditions:

*A factory produced 23,890 cars during the previous year. The factory shared their records, which verify this number, with a local journalist. The journalist reported that [24,000 / 23,933] cars were manufactured at this factory last year.*

Participants in the Approximate Condition learned that the journalist reported a rounded number (24,000), while participants in the Specific Condition learned that the journalist reported a non-rounded number (23,933). The number in the Specific Condition (23,933) was designed to be numerically closer to the ground truth (23,890) than the number in the Approximate Condition (24,000).

After reading the stimulus, participants first responded to a trust measure asking, “How much would you trust a report from this journalist about a different topic?” (1 = completely trust; 6 = completely distrust). Participants then responded to a truth classification measure asking, “The journalist said ‘[24,000 / 23,933] cars were produced last year.’ Is this true or false?” (True = 1, False = 0), where the bracketed information matched the stimulus participants had just read. Then, in an open-response question on the following page of the survey, participants reported why they classified the information from the journalist as true or false. Finally, participants answered the question, “What percentage of other people do you think would also say the journalist’s report is [true / false]?” (sliding scale, 0-100), where the bracketed information matched their response to the truth measure.

As in Studies S2A and S2B, we predicted that participants would be more likely to classify the information reported by the journalist as true in the Approximate Condition versus the Specific Condition.

### ***Results***

#### **Preregistered analyses**

To test whether participants would be more likely to classify the information reported by the journalist as true in the Approximate Condition versus the Specific Condition, we ran a generalized linear regression, predicting truth classification as a function of condition.

***Truth classification.*** We did not observe a significant effect of condition, *b* = 0.22, *SE* = 0.24, *z* = 0.92, *p* = 0.358.


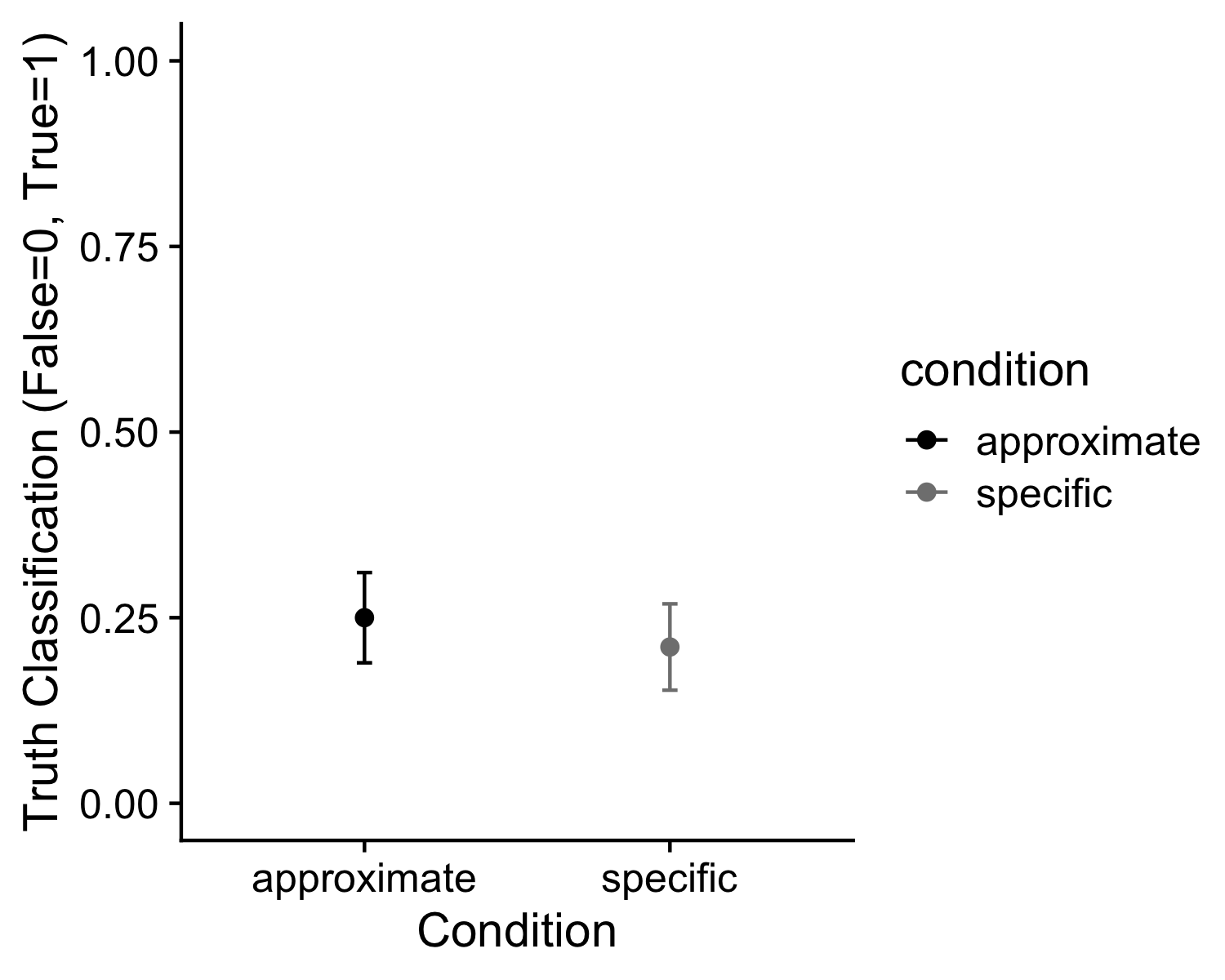


**Fig. S8.** Mean truth classifications, by condition (N=386). Error bars represent 95% confidence intervals.

## **Study S3**

### ***Methods***

#### **Participants**

802 U.S. participants were recruited on Amazon Mechanical Turk. We preregistered that we would collect 800 participants. Although we posted a HIT on Amazon Mechanical Turk for 800 participants, 802 ended up fully completing the study. Per our preregistered exclusion criteria, 58 participants were excluded for failing the pre-manipulation attention check, not completing the primary dependent measure, and/or taking the study more than once. Our final sample size was 744 (52.3% female, 46.8% male, 0.5% non-binary/other, 0.4% undisclosed; *M_age_* = 42.85).

#### **Materials and procedures**

This study employed a 2x2, between-subjects design. Each participant read the following stimulus, where the two bracketed sections indicate the information that was manipulated:

*463 people attended a local politician’s campaign rally. This number was verified by the number of tickets scanned at the door. The rally venue shared these verified numbers with a journalist. The journalist, who was trying to [accurately inform the public / inflate the politician's popularity], reported that [499 / 500] people attended the rally.*

Participants in the Inform Condition learned that the journalist was trying to “accurately inform the public” while those in the Deceive Condition learned that the journalist was trying to “inflate the politician's popularity.” Meanwhile, those in the Approximate Condition and the Specific Condition learned that the journalist reported that “500 people” and “499 people” attended the rally, respectively.

After reading the stimulus, participants first completed a trust measure and truth measure (in randomized order). The trust measure asked, “How much would you trust a report from this journalist about a different topic?” (1 = Completely distrust, 6 = Completely trust). We tested two different versions of the truth measure, which were counterbalanced across participants such that each participant responded to one version or the other. The first version (the “would you consider” version) asked, “Would you consider the journalist’s report that [499/500] people attended the rally to be true or false?” (1 = True, 0 = False) and the second version (the “is” version) asked, “Is the journalist’s report that [499/500] people attended the rally true or false?” (1 = True, 0 = False). We tested these two phrasings of the truth measure because we had employed the “is” phrasing in Study S3, but, in retrospect, were concerned that Amazon Mechanical Turk participants may have interpreted this as an attention check, and thus supplied the answer that they thought we as researchers would consider correct.

After completing the trust and truth measures, participants completed five measures about the journalist’s intentions. ​​The first four intent questions were asked in a matrix-style format that posed the question, “How much do you think the journalist was trying to do each of the following?” (1 = Not at all, 5 = A great deal), with the four response prompts being “Tell the truth to the public,” “Deceive the public,” “Report a precise number of rally attendees,” and “Report an approximate number of rally attendees.” The final intent question asked, “How morally good or morally bad would you consider the journalist’s intentions to be?” (1 = Extremely bad, 7 = Extremely good). Then, in an open-response question, participants reported why they classified the information from the journalist as true or false.

As preregistered, we predicted that participants would be more likely to judge the information provided by the journalist to be true in the Inform Condition versus the Deceive Condition and in the Estimate Condition versus the Specific Condition.

### ***Results***

#### **Primary analyses (preregistered)**

***Truth classifications.*** As preregistered, to assess the effects of our manipulations on truth classifications, we ran a logistic regression predicting truth classifications (collapsed across type of truth measure) as a function of the deceive-inform intent manipulation (effect-coded: 0.5 = inform; -0.5 = deceive), the specific-approximate report manipulation (effect-coded: 0.5 = approximate; -0.5 = specific), and the interaction between the manipulations. We observed a significant effect of the deceive-inform intent manipulation, *b* = 0.59, *SE* = 0.29, *z* = 2.03, *p* = 0.0427, such that participants were more likely to classify the claim as false in the Deceive Condition, all else equal. We observed a significant effect of the specific-approximate report manipulation, *b* = 1.52, *SE* = 0.29, *z* = 5.23, *p* < 0.001, such that participants were more likely to classify the claim as true in the Approximate Condition, all else equal. Finally, we observed a significant interaction effect between the two manipulations, *b* = -1.28, *SE* = 0.58, *z* = -2.20, *p* = 0.028, such that the effect of the deceive-inform intent manipulation was smaller in the Approximate Condition.


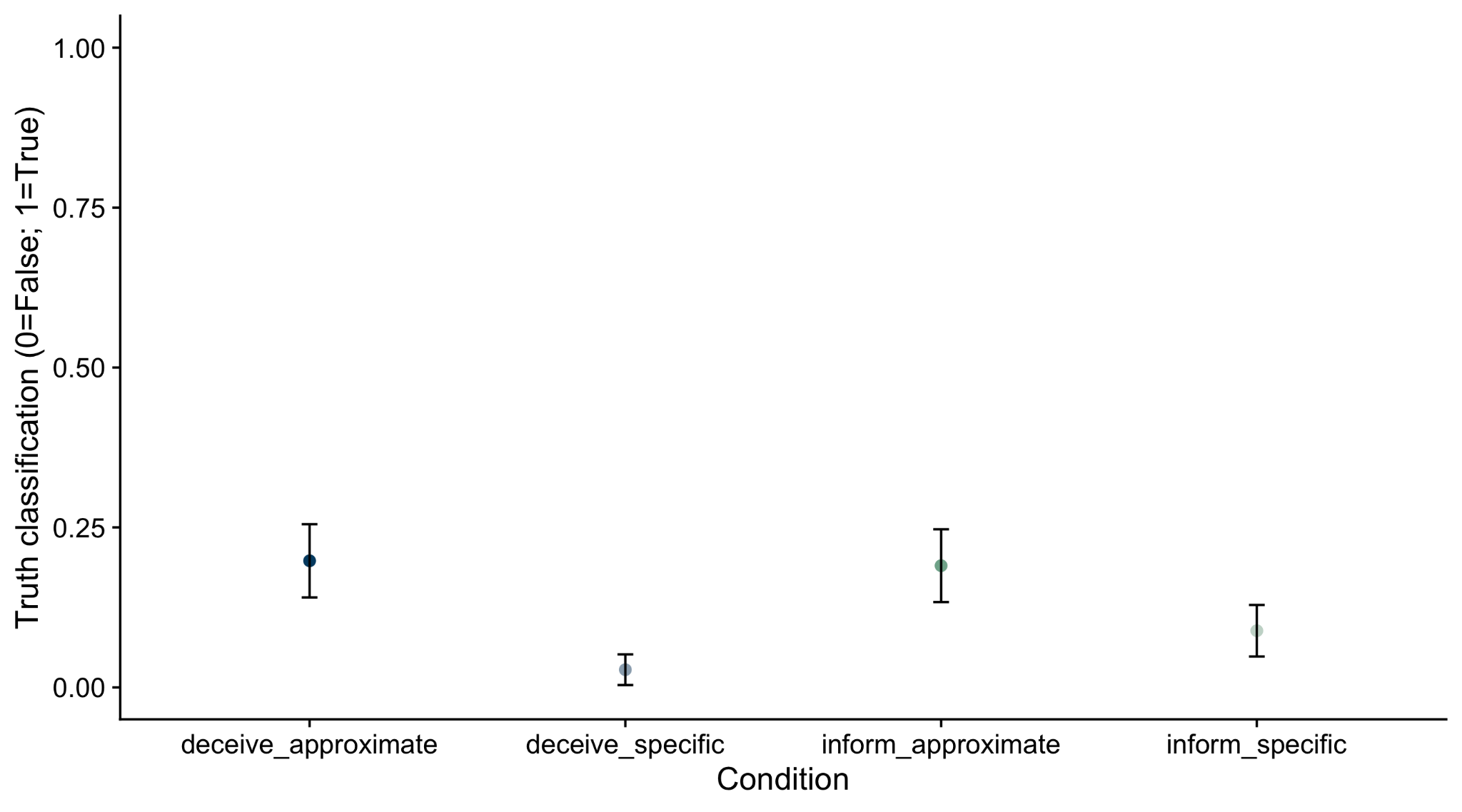


**Fig. S9.** Mean truth classifications, by condition (N=744). Error bars represent 95% confidence intervals.

#### **Secondary analyses (preregistered)**

***Trust judgments.*** As preregistered, we ran a linear regression predicting trust judgments as a function of the deceive-inform intent manipulation (effect-coded: 0.5 = inform; -0.5 = deceive), the specific-approximate report manipulation (effect-coded: 0.5 = approximate; -0.5 = specific), and the interaction between the manipulations. We observed a significant effect of the deceive-inform intent manipulation, *b* = 0.56, *SE* = 0.08, *t* = 6.68, *p* < 0.001, such that trust judgments were lower in the Deceive Condition, all else equal. We observed a significant effect of the specific-approximate report manipulation, *b* = 0.63, *SE* = 0.08, *t* = 7.61, *p* < 0.001, such that trust judgments were higher in the Approximate Condition, all else equal. Finally, we observed a significant interaction effect between the two manipulations, *b* = -0.538, *SE* = 0.17, *t* = -3.23, *p* = 0.001, such that the effect of the deceive-inform intent manipulation was smaller in the Approximate Condition.

***Correlation between truth classifications and trust judgments by condition.*** As preregistered, we tested the correlation between truth classifications and trust judgments within each condition. These values are reported in Table S15.

**Table S15. Study S3 correlations between truth and accuracy, by condition**

| **Condition** | **Correlation between truth and trust** |
| --- | --- |
| inform-approximate | *r* = 0.54 |
| inform-specific | *r* = 0.39 |
| deceive-approximate | *r* = 0.50 |
| deceive-specific | *r* = 0.32 |

***Truth measure wording.*** We preregistered that we would run a linear regression to test whether the wording of the truth measure influenced truth classifications. However, because the truth measure is dichotomous (“true” / “false”), a logistic regression would be more appropriate. Here, we report the results from both tests. In the linear regression, we found a significant effect of the truth measure wording, *b* = -0.079, *SE* = 0.02, *t* = -3.27, *p* = 0.001, such that participants were more likely to classify the claim as true when the truth measure asked “Would you consider the journalist’s report that [499/500] people attended the rally to be true or false?” versus “Is the journalist’s report that [499/500] people attended the rally true or false?” Similarly, in the logistic regression, we found a significant effect of the truth measure wording, *b* = -0.74, *SE* = 0.23, *t* = -3.20, *p* = 0.001, such that participants were more likely to classify the claim as true when the truth measure asked “Would you consider the journalist’s report that [499/500] people attended the rally to be true or false?” versus “Is the journalist’s report that [499/500] people attended the rally true or false?”

***Intent judgments.*** To assess the effects of our manipulations on each of our five measures of intent, we ran five linear regression models, each with one measure of intent as the dependent variable and three predictors: the deceive-inform intent manipulation (effect-coded: 0.5 = inform; -0.5 = deceive), the specific-approximate report manipulation (effect-coded: 0.5 = approximate; -0.5 = specific), and the interaction between the manipulations.

For intent-to-deceive judgments, we observed a significant effect of the deceive-inform intent manipulation, *b* = -1.07, *SE* = 0.09, *t* = -12.16, *p* < 0.001, such that intent-to-deceive judgments were higher in the Deceive Condition, all else equal. Additionally, we observed a significant effect of the specific-approximate report manipulation, *b* = -0.70, *SE* = 0.09, *t* = -7.99, *p* < 0.001, such that intent-to-deceive judgments were lower in the Approximate Condition, all else equal. Finally, we observed a significant interaction between the two manipulations, *b* = 0.43, *SE* = 0.18, *t* = 2.46, *p* = 0.014, such that the effect of the deceive-inform intent manipulation on intent-to-deceive judgments was smaller in the Approximate Condition, all else equal.

For the intent-to-tell-the-truth judgments, the results were consistent with those for the intent-to-deceive judgments. We observed a significant effect of the deceive-inform intent manipulation, *b* = 0.68, *SE* = 0.07, *t* = 9.31, *p* < 0.001, such that intent-to-tell-the-truth judgments were lower in the Deceive Condition, all else equal. We observed a significant effect of the specific-approximate report manipulation, *b* = 0.36, *SE* = 0.07, *t* = 4.91, *p* < 0.001, such that intent-to-tell-the-truth judgments were higher in the Approximate Condition, all else equal. Finally, we observed a significant interaction between the two manipulations, *b = -0.42, SE = 0.15, t = -2.85, p* = 0.004, such that the effect of the deceive-inform intent manipulation on intent-to-tell-the-truth judgments was smaller in the Approximate Condition, all else equal.

For intent-to-approximate judgments, we observed a significant effect of the deceive-inform intent manipulation, *b = 0.66, SE = 0.10, t = 6.77, p* < 0.001, such that intent-to-approximate judgments were lower in the Deceive Condition, all else equal. We observed a significant effect of the specific-approximate report manipulation, *b = 1.05, SE = 0.10, t = 10.81, p* < 0.001, such that intent-to-approximate judgments were higher in the Approximate Condition, all else equal. We did not find a significant interaction effect between the two manipulations, *b = -0.02, SE = 0.19, t = -0.12, p* = 0.902.

For intent-to-be-precise judgments, we observed a significant effect of the deceive-inform intent manipulation, *b = 0.33, SE = 0.07, t = 4.83, p* < 0.001, such that intent-to-be-precise judgments were lower in the Deceive Condition, all else equal. We observed a significant effect of the specific-approximate report manipulation, *b = -0.28, SE = 0.07, t = -4.05, p* < 0.001, such that intent-to-be-precise judgments were higher in the Specific Condition, all else equal. Finally, we observed a significant interaction effect, *b = -0.47, SE = 0.14, t = -3.41, p* < 0.001, such that the effect of the specific-approximate report manipulation was smaller in the Deceive Condition relative to the Inform Condition.

For moral intent judgments, we observed a significant effect of the deceive-inform intent manipulation, *b =* 0.69*, SE =* 0.08*, t =* 8.23*, p* < 0.001, such that moral intent judgments were lower in the Deceive Condition, all else equal. We observed a significant effect of the specific-approximate report manipulation, *b =* 0.58*, SE =* 0.08*, t =* 6.93*, p* < 0.001, such that moral intent judgments were higher in the Approximate Condition, all else equal. Finally, we observed a significant interaction between the two manipulations, *b =* -0.66*, SE =* 0.17*, t =* -3.96*, p* < 0.001, such that the effect of the deceive-inform intent manipulation on moral intent judgments was smaller in the Approximate Condition, all else equal.

## **Study S4**

The data in Study S4 come from a large survey conducted on people’s beliefs about the concept of truth. Embedded in this survey was an experiment that tested the same deceptive-intent manipulation as that employed in Study 2 (see main text), but also varied the instructions such that one group of participants believed that there were no right or wrong answers, while another group of participants believed that some answers were more correct than others. Additionally, this survey directly asked participants about the role intent plays in their truth classifications.

The rest of the data from this survey will be reported elsewhere because they concern many different truth-related topics; the full survey and dataset can be accessed on this project’s OSF page (https://osf.io/d3wa8/?view_only=be0a929b2f154a71bae6ce2d3a7836bb). Study S4 was not preregistered.

### ***Methods***

#### **Participants**

1,000 U.S. participants were recruited on Amazon Mechanical Turk via the CloudResearch-Approved Participants List. Before signing the consent form, participants were told, “The questions in this study require a fair amount of thought, and a few require open-ended written responses. Please only participate if you are willing to think deeply about all of the questions you will be asked.” 120 participants were excluded for failing one or both attention checks, not completing all dependent measures, taking the study more than once, and/or completing the study in less than 50% of the median completion time. Our final sample size was 880 (43.9% female, 54.5% male, 0.5% non-binary/other, 1.1% undisclosed; *M_age_* = 42.84).

#### **Materials and procedures**

At the beginning of the survey, participants were randomly assigned to receive one of two different sets of instructions. The Personal Beliefs Instructions stated, “We’re interested in how people think about truth. There are no right or wrong answers to the questions in this survey. Please give your honest opinions about what you think truth is and what kinds of things you think are true—not what you think others believe.” The Knowledge Assessment Instructions stated, “We’re interested in how people think about truth. According to philosophers who study truth, there are more correct and less correct answers to the questions in this survey. We want to know how well people score on questions about what truth is and what kinds of things are true.”

After responding to other questions about truth (which can be viewed on this project’s OSF page), participants read two stimuli. These stimuli were very similar to those employed in Study 2, except they did not contain the specific-approximate report manipulation (see table S16).

Each stimulus could be shown in one of two conditions. In the Inform Condition, the information source in the stimulus was said to be trying to accurately inform their audience. In the Deceive Condition, the information source was said to be trying to deceive their audience. For example, one stimulus read, “The mayor of a large city was informed that lead levels in the city water were at a dangerous level of 113 µg/L. During a press conference, the mayor, trying to [deceive the public / accurately inform the public] about the state of the city water, reported that the lead levels were 100 μg/L.” The portion in brackets varied by condition. Each participant saw one stimulus in the Deceive Condition and one in the Inform Condition. We varied which stimulus was assigned to which condition, and the order in which each condition and stimulus were presented. Because the information source reported a rounded number in both stimuli (see table S16), the Deceive Condition was equivalent to the Deceive-Approximate Condition in Study 2 and the Inform Condition was equivalent to the Inform-Approximate Condition in Study 2 (see main text).

After each stimulus, participants answered a single question: “Would you consider the report from [*information source*] to be true or false?” (True; False; Unsure). After reading and responding to both stimuli, participants were then asked, “Imagine someone said something that was quite close to the truth, but was not perfectly accurate. Would whether that person was trying to deceive their audience on purpose influence how you classify the truth of their statement?” (Yes; No; I don’t understand the question; I understand the question, but I’m not sure).

The instructions manipulation at the beginning of the study was designed to prompt people to think about the questions in our study in a more objective fashion (Knowledge Assessment Instructions) or more subjective fashion (Personal Beliefs Instructions). Because the objective accuracies of the claims in this study were both fully known by participants and unchanged between conditions, we reasoned that framing the task as having correct and incorrect answers would lead people to focus more on the objective discrepancy between the claim and the ground truth, and thus be less likely to show the previously observed effect of informative versus deceptive intent. In short, we expected the Knowledge Assessment Instructions condition to be a more stringent test of our hypothesis that the intentions of the information source affect people’s truth classifications, even when people know the ground truth. Specifically, we hypothesized that participants would be less likely to rate the information source’s claim as true when the information source was said to have deceptive intent versus informative intent (which would replicate the comparison between the Deceive-Approximate Condition and ​​the Inform-Approximate Condition from Study 2), but that this effect would be smaller when participants received the Knowledge Assessment Instructions versus the Personal Beliefs Instructions. We did not have a strong hypothesis about the question asking participants whether intent would influence their truth classifications.

**Table S16. Study S4 Stimuli**

| **Stimulus number** | **Stimulus** |
| --- | --- |
| 1 | A journalist learned that 463 people attended a local politician’s campaign rally. The journalist, trying to [accurately inform / deceive] the public, reported that 500 people attended the rally. |
| 2 | The mayor of a large city was informed that lead levels in the city water were at a dangerous level of 113 µg/L. During a press conference, the mayor, trying to [accurately inform / ​​deceive] the public about the state of the city water, reported that the lead levels were 100 μg/L. |

The two stimuli used in Study S4. The bracketed sections represent the information that was manipulated depending on the condition in which participants saw the stimulus. In the Inform Condition, the information source was said to be trying to accurately inform their audience, while in the Deceive Condition the information source was said to be trying to deceive their audience.

### ***Results***

**Truth classifications.** We ran a generalized linear mixed effects model predicting truth classifications as a function of the deceive-inform intent manipulation (effect-coded: Inform Condition = 0.5; Deceive Condition = -0.5), the instructions manipulation (effect-coded: Personal Beliefs Instructions = 0.5; Knowledge Assessment Instructions = -0.5), and the interaction between the two, with random intercepts for participants. Before running this model, we dropped the relatively few participants (n=49) who answered the truth classification question to either stimulus as “unsure,” leaving a sample size of n=831. We found a significant effect of the deceive-inform intent manipulation, *b* = 3.91 (OR = 49.88, OR 95% CI [18.53 – 134.24]), *SE* = 0.51, *z* = 7.74, *p* < 0.001, such that participants were less likely to classify the information source’s report as true when the information source was said to be trying to deceive (versus inform) their audience. We did not find a significant effect of either the instructions manipulation, *b* = 0.03 (OR = 1.03, OR 95% CI [0.31 – 3.44]), *SE* = 0.62, *z* = 0.05, *p* = 0.962, or the interaction between the deceive-inform intent manipulation and the instructions manipulation, *b* = 0.20 (OR = 1.22, OR 95% CI [0.26 – 5.66]), *SE* = 0.78, *z* = 0.25, *p* = 0.799. See Figure S10, panel (a).

**Beliefs about intent and truth classifications.** For the measure asking participants to explicitly report whether an intent to deceive would influence their truth classifications, 57.7% (508/880) of the total sample answered “yes,” 36.8% (324/880) answered “no,” 0.6% (5/880) answered “I don’t understand the question,” 4.9% (43/880) answered “I understand the question, but I’m not sure.” To test whether the instructions manipulation affected the proportion of participants responding “yes” versus the proportion responding “no” to this measure, we ran a logistic regression predicting yes/no responses as a function of the instructions provided (N=832). We did not find a significant difference in yes/no responses between participants who saw the Personal Beliefs Instructions compared to those who saw the Knowledge Assessment Instructions, *b* = 0.08 (OR = 1.09, OR 95% CI [0.82 – 1.43]), *SE* = 0.14, *z* = 0.58, *p* = 0.564. See Fig. S10, panel (b).


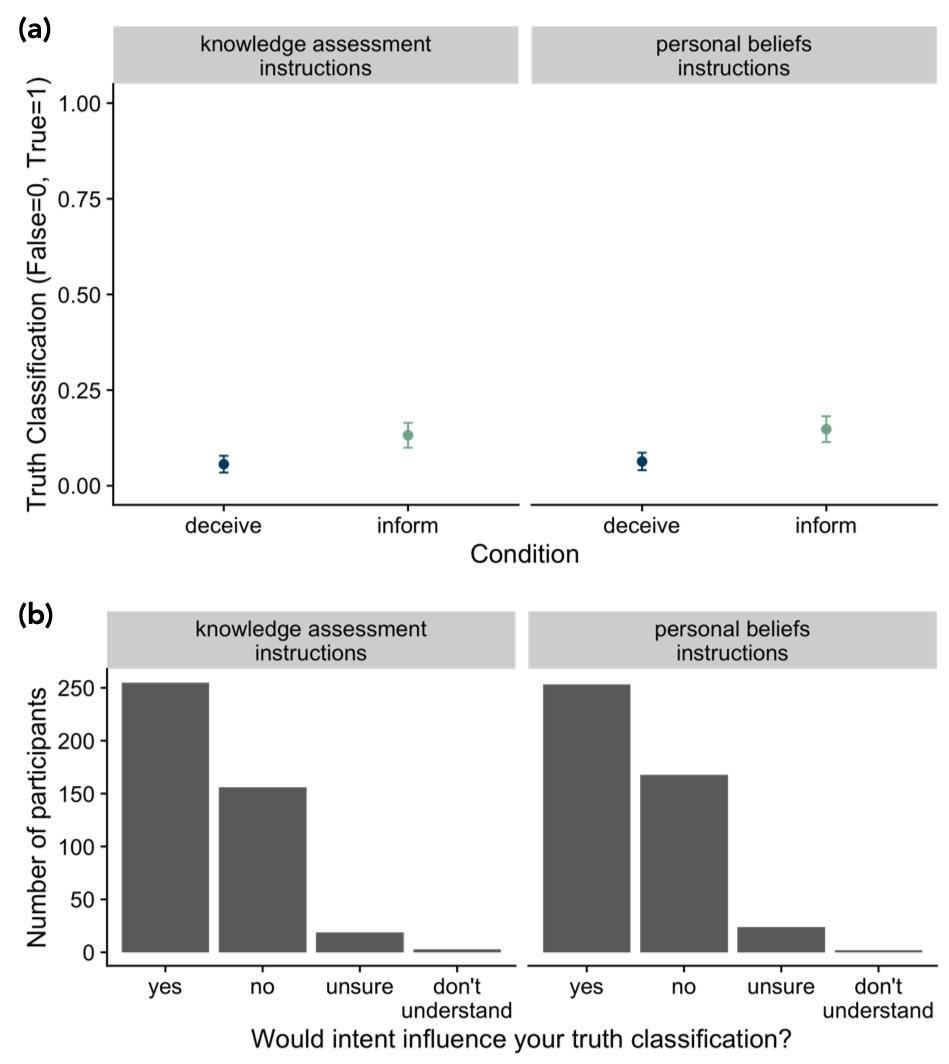


**Fig. S10.** Results from Study S4. (a) Mean truth classifications broken down by the deceive-inform intent manipulation and the instructions manipulation (N=831). Error bars represent 95% confidence intervals. (b) Frequency of each response to the beliefs-about-intent-and-truth-classifications question split by the instructions manipulation (N=880).

### ***Discussion***

Study S4 found that participants were more likely to classify a claim as false when the information source was said to have deceptive (versus informative) intent, even though the discrepancy between the claim and the ground truth was known by participants and did not change between conditions. We found no evidence that the effect of deceptive intent on truth classifications differed when people thought they were merely providing their opinion (Personal Beliefs Instructions) versus when they thought their responses would be evaluated relative to established standards of truth (Knowledge Assessment Instructions).

Additionally, a majority of participants (57.7%) reported that whether someone was trying to deceive their audience on purpose would influence whether they would classify that person’s statement as true or false. We did not find evidence that the proportion of participants anticipating that intent would influence their truth classifications depended on whether participants saw the Personal Beliefs Instructions or the Knowledge Assessment Instructions.

We believe the following three findings from Study S4 could suggest that at least some participants have introspective access to the role intent attributions play in their truth classifications: (1) more than half of Study S4 participants reported that intent would influence their truth classifications; (2) intent influenced participants’ truth classifications in a within-subjects design in which it was explicitly stated in one case that the information source was trying to inform their audience and in the other case that the information source was trying to deceive their audience (as was the case in Study 2, as well); and (3) we did not find evidence that either of these effects was influenced by whether participants thought they were being evaluated against established standards of truth.

# **References**

Bates, D., Kliegl, R., Vasishth, S., & Baayen, H. (2018). Parsimonious Mixed Models. *arXiv*. Retrieved from http://arxiv.org/abs/1506.04967

Grice, J. W., Medellin, E., Jones, I., Horvath, S., McDaniel, H., O’lansen, C., & Baker, M. (2020). Persons as effect sizes. *Advances in Methods and Practices in Psychological Science*, *3*(4), 443-455. https://doi.org/10.1177/2515245920922

Matuschek, H., Kliegl, R., Vasishth, S., Baayen, H., & Bates, D. (2017). Balancing Type I error and power in linear mixed models. *Journal of Memory and Language, 94*, 305–315. https://doi.org/10.1016/j.jml.2017.01.001

McManus, R. M., Young, L., & Sweetman, J. (2023). Psychology is a feature of persons, not averages or distributions: The group-to-person generalizability problem in social cognition research. *Manuscript under review*. https://moralitylab.bc.edu/wp-content/uploads/2022/05/AMPPS-22-0036_Proof_hi.pdf
